# Morpho-physiological adaptations of *Leptocylindrus aporus* and *L. hargravesii* to phosphate limitation in the northern Adriatic

**DOI:** 10.1038/s41598-022-06062-5

**Published:** 2022-02-17

**Authors:** Nataša Kužat, Daniela Marić Pfannkuchen, Mirta Smodlaka Tanković, Ana Baričević, Ingrid Ivančić, Ivna Vrana, Blaženka Gašparović, Martin Pfannkuchen

**Affiliations:** 1 Center for Marine Research, Ruđer Bošković Institute, Rovinj, Croatia; 2 Division for Marine and Environmental Research, Ruđer Bošković Institute, Zagreb, Croatia

**Keywords:** Ecology, Ecology, Environmental sciences, Ocean sciences

## Abstract

The northern Adriatic is highly productive and shallow area characterized by numerous spatio-temporal gradients (e.g. nutrients, salinity, temperature). It is strongly influenced by numerous freshwater inputs, mainly from Po river. Its current systems as well as Po river, generates gradients of phosphate availability with an expressed N/P imbalance and phosphate limitation. A number of recent studies characterized these gradients as major factors affecting abundance and composition of microphytoplankton communities. Focus of this study is on two *Leptocylindrus* species, *Leptocylindrus aporus* (F.W. French & Hargraves) D. Nanjappa & A. Zingone 2013 and *Leptocylindrus hargravesii* D. Nanjappa & A. Zingone 2013. Species belonging to *Leptocylindrus* genus are frequently observed and have high abundances and also high contributions to the microphytoplankton community in this area. We focused on their morphological and physiological responses to phosphate limitation in situ and also performed in vitro experiments. In this study we report data on species specific growth rates under phosphorus (P) deplete and P rich conditions, localization and characteristics of alkaline phosphate activity, phosphate uptake rates as well as their morphological differences in P deplete versus P rich conditions. Our in vitro experiments showed that both *Leptocylindrus* species morphologically reacted similarly to phosphorus depletion and showed significantly elongated pervalvar axis in P depleted conditions if compared to P rich conditions. Also average chain lengths increased when in P depleted conditions. Two previously mentioned adaptations indicate their tendency to increase cellular surface areas available for alkaline phosphatase. Chlorophyll fluorescence of both species significantly decreased in P depleted medium. Although both species morphologically reacted similarly, our experiment demonstrated significant differences in physiological reactions to P depleted conditions.

## Introduction

*Leptocylindrus* Cleve, 1889 (Bacillariophyta, Leptocylindraceae) is a genus of centric diatoms. It comprises mostly cosmopolitan species, that are major components of marine coastal phytoplankton communities worldwide and that can be major contributors to diatom blooms^1^. In the past, two species were considered to belong to this genus, *Leptocylindrus danicus* Cleve 1889, and *L. minimus* Gran 1915. A third species currently in the genus *Leptocylindrus* is *L. mediterraneus* (H. Peragallo) Hasle 1975 but its taxonomic position has been questioned^2^. Recent molecular phylogenetic analysis suggests that it should no longer belong to genus *Leptocylindrus* but rather to the genus *Dactyliosolen*^3^. In the year 2013 two new species were described, *L. convexus* D. Nanjappa & A. Zingone 2013 and *L. hargravesii* D. Nanjappa & A. Zingone 2013. Furthermore, *Leptocylindrus danicus* var. *apora* F.W. French & Hargraves 1986 was raised to species level as *L. aporus* (F.W. French & Hargraves) D. Nanjappa & A. Zingone 2013^1^. The number of species within the genus *Leptocylindrus* hence has in recent years increased from 2 (or 3, including *L. mediterraneus*) to 5 (or 6) species.

The morphology of *Leptocylindrus* species is rather simple. Cells are narrow, long and cylindrical, they can be connected by valve faces to form tight filamentous chains but also can be solitary. Cells contain various numbers of plastids which are of variable shape^1^. Their simplicity results in significant difficulties for the identification (to species level) by light microscopy. Often molecular methods and electron microscopy should be employed for species identification.

The northern Adriatic Sea is the northernmost area of the Mediterranean. It is a highly productive, shallow, marine ecosystem that is characterized by steep spatio-temporal ecological gradients^4,5^. The productivity of the northern Adriatic is determined by numerous freshwater inflows^6^, most importantly from the Po river. The Po river is the largest freshwater input into the Mediterranean delivering freshwater from the industrial and agronomical waters from the highly developed northern part of Italy^7^. The West Adriatic Current (WAC) transfers nutrients from north to south Adriatic along the western coast of the Adriatic Sea^8^. There are also situations when the fresh water inflow is limited^9^, and such events have a significant impact on the composition of phytoplankton communities in this area. In addition to river dilution, there are periodic inflows of high salinity waters transferred by the Eastern Adriatic Current (EAC) from the southern to the northern part of the Adriatic Sea^10^. Consequently, steep and diverse spatio-temporal ecological gradients are formed, along which a diatom dominated microphytoplankton community can be observed and studied in situ^11^. A number of recent studies characterized gradients of phosphate availability as major drivers of microphytoplankton communities (abundance and composition)^5,12,13^. Some phytoplankton species are highly sensitive to phosphorus (P) availability, which affects physiological performance and biochemistry (e.g. photosynthetic efficiency, biovolume, growth…). It also affects the composition and abundance of marine phytoplankton communities^4,14,15^. Our earlier results showed that the phytoplankton behaviour and growth is mainly determined by temperature, light and capability of species to cope with ever-changing phosphorus availability that includes times of inorganic P deficiency^4,13^. In the absence of inorganic phosphorus, diatoms such as *Chaetoceros peruvianus*^5,12^ can use organic phosphorus to survive in the environment. One of the mechanisms for the utilization of organic phosphorus from the environment is the synthesis of extracellular alkaline phosphatase (AP) that dephosphorylates dissolved organic phosphorus compounds such as phosphosugars, nucleic acids, and phospholipids^16^ and makes the resulting phosphates available to cells^17^. Next to the mechanisms mentioned above, diatom species also developed additional strategies that are helping them to survive/compete in P-limited conditions. When stressed, many diatoms accumulate lipids. Lipid accumulation is part of a general response to nutrient stress whereby the metabolism is shifted from biomass production to energy storage^18^. Phospholipids are prominent building blocks of all cellular membranes. In P depleted conditions, cells have developed the ability to replace phospholipids with non-phospholipids resulting in a reduced phospholipid content of the cellular membranes^19–21^. Limited availability of inorganic phosphorus can additionally affect the synthesis of pigments. It is assumed that when phosphorus is deficient, pigment synthesis stops because cells are no longer able to synthesize and transcribe RNA, resulting in reduced cellular concentrations of chlorophyll *a*^22–26^. Finally some diatoms significantly change their morphology (elongated cells, thicker setae) in P limited conditions^12^. It is assumed to be a consequence of the abovementioned physiological reactions and appears to serve as improved adaptation and competition for P in depleted conditions.

Altogether, numerous phytoplankton species, when limited by inorganic P have developed numerous adaptations to compete for limiting nutrients and to survive longer periods of unfavourable conditions. Detailed studies of physiological adaptations at species level almost always require in vitro experimental approaches that are used to isolate chosen environmental conditions, to identify trigger mechanisms and whose results have to be verified against in situ observations^12^. Here we focus on two *Leptocylindrus* species, *L. aporus* and *L. hargravesii.* Both are new to the Adriatic Sea and one of them is only recently described*.* Here, we represent experimental data and in situ observations for the two mentioned species. We report data on species specific growth rates under different nutrient regimes, phosphate uptake rates, alkaline phosphatase activity, localization and activation patterns and characteristics of alkaline phosphatase activity, P uptake dynamics and changes in the lipid composition as well as their morphological reactions to phosphate limited conditions.

## Materials and methods

### Sampling and establishment of monoclonal cultures

**Figure 1:**
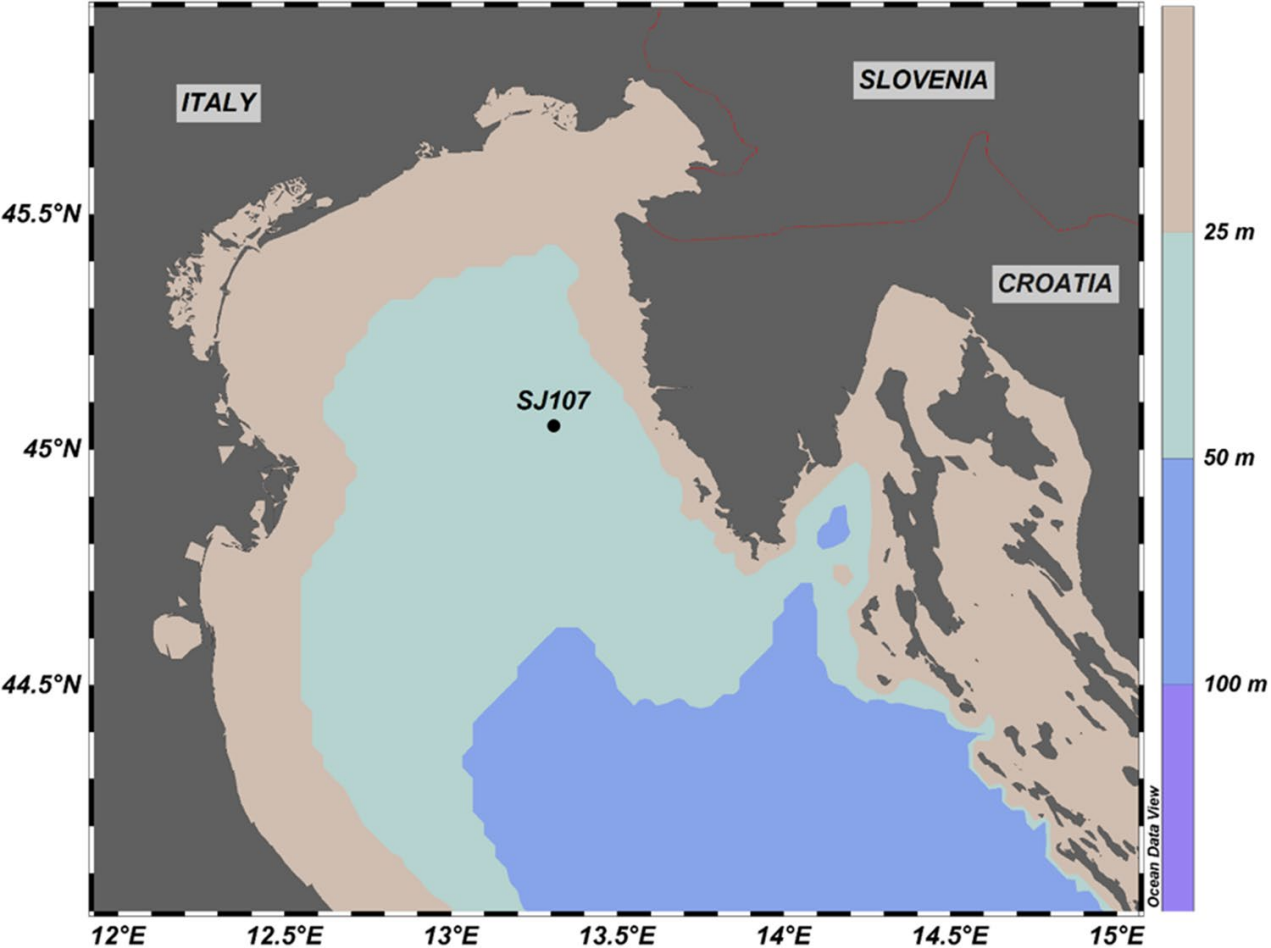
Sampling area: station (SJ107) where vertical net hauls for isolation of the species were performed is marked.

Samples for the isolation of *Leptocylindrus* species were taken in the northern Adriatic Sea at station SJ107 (latitude: 45° 05N, longitude: 13° 31E) (Fig. 1). Sampling was conducted by phytoplankton net (opening diameter 50 cm, length 2.50 m, mesh size 53 μm). Vertical net hauls were performed from 15 m of depth to the surface. *Leptocylindrus* cells were light microscopy identified and manually isolated with Pasteur pipettes from live net samples. Cultures were established by single cell or single chain isolation. Cells were grown in monoclonal batch cultures in F/2 medium^27^ in sterile 40 mL vented culture flasks and incubated at 16 °C and 75 μmol photons m^−2^ s^−1^ on 12:12 h light/dark photoperiod. *Leptocylindrus* monoclonal batch cultures were associated to Center for Marine Research, Rovinj, Culture Collection under numbers CIM 869 and CIM 874. Once cultures were healthy and reached exponential growth phase they were ready for molecular species identification and in vitro experimental phase.

Spatio-temporal distribution data were extracted from a long term phytoplankton in situ monitoring data set conducted by monthly to quarterly sampling between the years 1978 and 2020. Sampling was conducted following the methodology described earlier^11,13^.

### Quantitative phytoplankton community analysis

Phytoplankton samples (200 mL) used for quantitative analysis were fixed with neutralized formaldehyde (2% final concentration) and analyzed in 50 mL subsamples with an Axiovert 200 light invert microscope (Zeiss GmbH, Oberkochen, Germany) following the Utermöhl method^28^ as described in more detail earlier^13^.

### Experiments setup

Experiments followed the methodology described earlier^12^ with the following adjustments.

In vitro cultures were prepared by inoculation of 1 mL of monoclonal cultures CIM869 (*L. aporus)* or CIM874 (*L. hargravesii*) in 200 mL of two different media. Nutrient rich conditions were simulated by F/2 medium^27^ and dissolved inorganic P limitation stress was simulated by P-limited medium (F/2 medium without sodium hydrogen phosphate). Each culture condition was prepared and followed in triplicates. Both media were prepared with the Northern Adriatic seawater rested in the dark for 2 months. Seawater was filtered twice through MF-Millipore™ Membrane Filter, 0.22 µm pore size (Merck Millipore Ltd.) and boiled in a microwave oven^29^. Cultures were incubated in climate chamber (Memmert ICH110, Germany) with a light–dark cycle of 12:12 h in sterile 250 mL vented culture flasks (easy flasks, Nuclon, Denmark) at 16 °C and irradiance of 75 μmol photons m^−2^ s^−1^.

Batch cultures were followed through the course of the experiment (17 days). For each of the parameter described in the following paragraph three independent measurements of three independent cultures for each regime were performed and the given results are averages across triplicate measurements of triplicate cultures for the respective culture condition. For growth curve and chain length analysis, cell numbers of all three triplicate cultures were analyzed together and averaged.

### Growth dynamics and morphology

Cell concentrations and chain lengths were analyzed every second or third day in Sedgewick-Rafter counting chambers on a Zeiss Axiovert 200 microscope.

Morphological analysis was performed at the end of the exponential growth phase. Five subsamples of 20 μL for each culture were analysed by DAPI staining on a Zeiss Axioimager fluorescence microscope using the Zeiss filterset 49 for epifluorescence as well as bright field phase contrast for transillumination^30^. Only cultures with no bacterial sized (0.2–3 μm) and DAPI positive particles were further analyzed. Morphological analyzes were carried out using Zeiss Axiovert 200 light microscope (LM) (Carl Zeiss, Oberkochen, Germany) equipped with Nomarski differential interference contrast (DIC), phase contrast, and bright-field optics. Light micrographs were taken using a Zeiss Axiocam digital camera and all morphological measurements were made in software suite Axiovision 4.8 (ZEISS, Oberkochen, Germany). The terminology used to describe morphological features of *Leptocylindrus* species follows Anonymous^31^ and Ross et al.^32^. Biovolume of *Leptocylindrus* species was calculated using the following formula: V = π/4 * cell diameter^2 ^* cell height^33^.

### Molecular species identification

Two barcodes were used for molecular phytoplankton species identification: 5’ end region of the ribulose bisphosphate carboxylase large subunit (rbcL) and V4 region of the small subunit (18S) ribosomal RNA gene. CIM874 and CIM869 cell cultures (30 mL) were filtered on 1.2 µm cellulose filters (Merck Millipore) and frozen on − 80 °C until further processing. Genomic DNA was isolated with DNeasy Plant Mini Kit (Qiagen) according to manufacturer instructions and PCR amplifications conducted as described in Smodlaka Tankovic 2018^12^. RbcL barcode was PCR amplified using primer pair rbcL66 + (5′-TTAAGGAGAAATAAATGTCTCAATCTG-3′) and DtrbcL3R (5′-ACACCWGACATACGCATCCA-3′)^34,35^. 18S barcode was amplified using primer pair D512 (5′-ATTCCAGCTCCAATAGCG-3′) and D978 (5′-GACTACGATGGTATCTAATC-3′)^36^. PCR products were sequenced at Macrogen Europe (The Netherlands) and Geneious 7.1.7. software^37^ was used for sequence analyses. For each barcode a high quality (every nucleotide position confirmed with chromatogram quality) consensus sequence was generated and MAFFT program^38^ was used for multiple alignments with the available GenBank^39^ database sequences. Maximum likelihood method and substitution model Kimura was used for phylogenetic trees constructions with PhyML program^40^.

### Chlorophyll fluorescence intensity

Chlorophyll fluorescence intensity was measured every second or third day in black 96 well microplates. Samples were prepared in a dark environment. Measurements were conducted on microplate reader (Infinite M200Pro, Tecan GmbH, Austria) where we measured fluorescence intensity with excitation at 460 nm and emission at 685 nm. Triplicates of every culture were measured. Cellular chlorophyll *a* (CHLa) fluorescence was calculated as measured chlorophyll fluorescence divided by cell count of each sample.

### Alkaline phosphatase activity (APA) and subcellular localization

APA in vitro, as all other analyses was measured every second or third day in cultures as described earlier^5,12^. Substrate 4-methylumbelliferyl phosphate (MUF-P, Sigma Aldrich, Germany) was added to *L. aporus* and *L. hargravesii* cultures with final reaction volume of 250 μL and final concentration of 50 μmol l^−1^ h^−1^ (saturation concentration). Product concentrations were measured directly after the addition of the substrate and further after 10, 30 and 60 min of reaction time. Products were detected by fluorescence intensity on a Tecan M200 Pro spectrofluoremeter (excitation at 365 nm and emission at 460 nm). Standard curves were generated with concentrations ranging from 0.008 to 3 μM for 4-methylumbelliferyl (MUF, Sigma Aldrich, Germany). Kinetic parameters for the APA were determined at 15 substrate concentrations between 0.5 and 400 µM. Results were analyzed using non-linear fitting of the package Dcr in the software environment R^41^.

Localization of alkaline phosphatase activity was performed with the ELF_97 Endogenous Phosphatase Detection Kit (E6601) (Thermo Fisher Scientific, Waltham USA) as described earlier^5,42,43^. Live cells where incubated with the phosphatase substrate and analyzed with a Zeiss Axiovert Epifluorescence microscope. Chloroplasts were detected by their autofluorescence (Filterset 14 Zeiss), and insoluble fluorescent product of alkaline phosphatase activity was localized using a specially adapted filter set (excitation: 340/26, beamsplitter 400 longpass, emission 525/50).

### Dissolved phosphate

Samples for determining PO_4_ concentrations were prepared by adding 20 µl of mixed reagent (molybdate, sulphuric acid, citrate and tartarate)^44^ to 200 µl of cell cultures and the measurements were conducted on microplate reader (Infinite M200Pro, Tecan GmbH, Austria) where we measured absorbance at 889 nm. Triplicates of each culture and the standard curves of graded KH_2_PO_4_ solutions (concentrations ranging from 0.25 to 250 μM) were measured simultaneously. Cellular phosphate uptake was calculated as amount of phosphate removed from the culture medium between two measurements divided by either the cell numbers at time of the measurement (lower limit), or by the cell numbers at the time of the last measurement (upper limit).

### Lipid analysis

At the end of experiment (day 17), sample (80 mL) of each culture triplicate was filtered on precombusted 0.7 μm Whatman GF/F filters to determine the lipid composition of diatoms *L. aporus* and *L. hargravesii*. The filters were stored at − 80 °C until lipid extraction.

The lipid extraction was carried out by a modified Bligh and Dyer method^45^. To the sliced filters in the cuvettes we added 10 mL of monophasic dichloromethane:methanol:deionised water, 1:2:0.8 v/v/v solution and 10 μg of standard nonadecanone. It was then 3 min ultrasonicated, filtered through a sinter funnel into a separatory funnel, washed again with 10 ml of monophasic solution, and then with 5 ml dichloromethane and 5 ml 0.73% NaCl. The lipids in dichloromethane were collected and extraction was repeated once more with 10 ml of dichloromethane. N–nonadecanone was added as internal standard to each sample to estimate the recoveries in the subsequent steps of the sample analysis. Extracts were evaporated to dryness under nitrogen flow and dissolved in 20–50 μL dichloromethane (Merck, USA) (arbitrarily determined based on the researcher experience).

Total lipid and lipid class quantitation was performed by Iatroscan thin layer chromatography/flame ionization detection (TLC/FID) (Iatroscan MK-VI, Iatron, Japan) with a hydrogen flow of 160 ml min^−1^ and air flow of 2000 ml min^−1^. Eighteen lipid classes (hydrocarbons (HC), sterol esters (SE); fatty acid methyl esters (ME); fatty ketone nonadecanone (KET, internal standard); triacylglycerols (TG); free fatty acids (FFA); fatty alcohols (ALC); 1,3-diacylglycerols (1,3 DG); sterols (ST); 1,2-diacylglycerols (1,2 DG); pigments (PIG); monoacylglycerols (MG); mono- and di-galactosyldiacylglycerols (MGDG and DGDG); sulfoquinovosyldiacylglycerols (SQDG), phosphatidylglycerols (PG); phosphatidylethanolamines (PE); and phosphatidylcholine (PC)) were separated on Chromarods SIII and quantified by an external calibration with standard lipid mixture. For this work we elaborate cellular lipids including TG, SE, ST, PIG, phospholipids PG, PE and PC, glycolipids MGDG, DGDG, and SQDG. Sample aliquots (2 µL) in dichloromethane were spotted by semiautomatic sample spotter. Each lipid extract was analysed in duplicate. The standard deviation determined from duplicate runs accounted for 5–12% of the lipid classes' relative abundance. The separation scheme for all classes involved seven elution steps in the solvent systems of increasing polarity^46,47^.

### Statistical analyses

Growth curves were analyzed using non-linear fitting with the assumption of sigmoidal growth in batch cultures of the packages Growthcurver and Ggplot2 in software environment R^48–50^. Multiple analysis of variance was performed using the function MANOVA and t-tests (Welch’s Two Sample t-test) were performed using the function t-test of the R base package^49^. Several parameters were also analyzed using Excel 2013 software environment. Level of statistical significance for different parameters are shown with *p* value where significant statistical differences are demonstrated as values ≤ 0.05. Figure 1 was drawn with Ocean Data View (ODV) software^51^.

## Results

### *Leptocylindrus* cultures species identification

**Figure 2:**
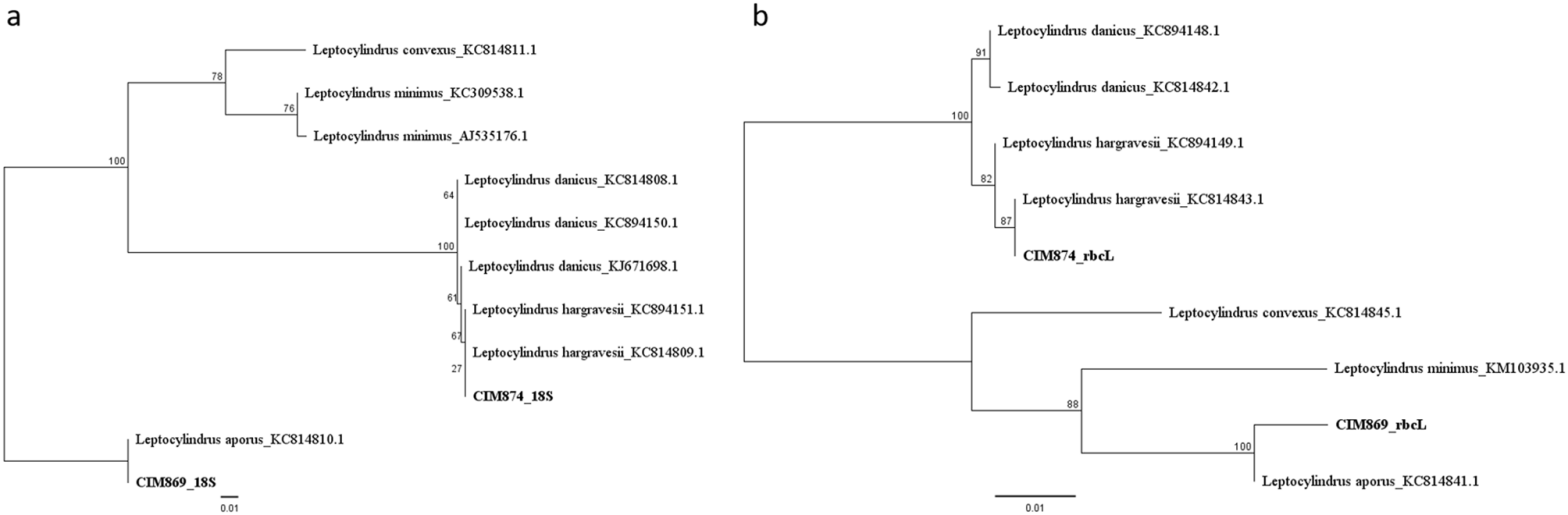
*Leptocylindrus* barcodes 18S (**a**) and rbcL (**b**) phylogenetic reconstruction with CIM cultures and GenBank sequences. For each GenBank sequence species and accession number are indicated. CIM culture sequences are marked in bold. Bootstrap values are indicated next to tree nodes.

*Leptocylindrus* cultures CIM869 and CIM874 were taxonomically identified with light microscopy and DNA barcoding as *L. aporus* and *L. hargravesii* respectively. Sequences were deposited in GenBank under Accession numbers: MW704023-MW704024 and MW715667-MW715668. Phylogenetic reconstructions of the *Leptocylindrus* genus for the two used barcodes are presented in Fig. 2.

### Temporal and spatial distribution of *Leptocylindrus* sp. Species

**Figure 3:**
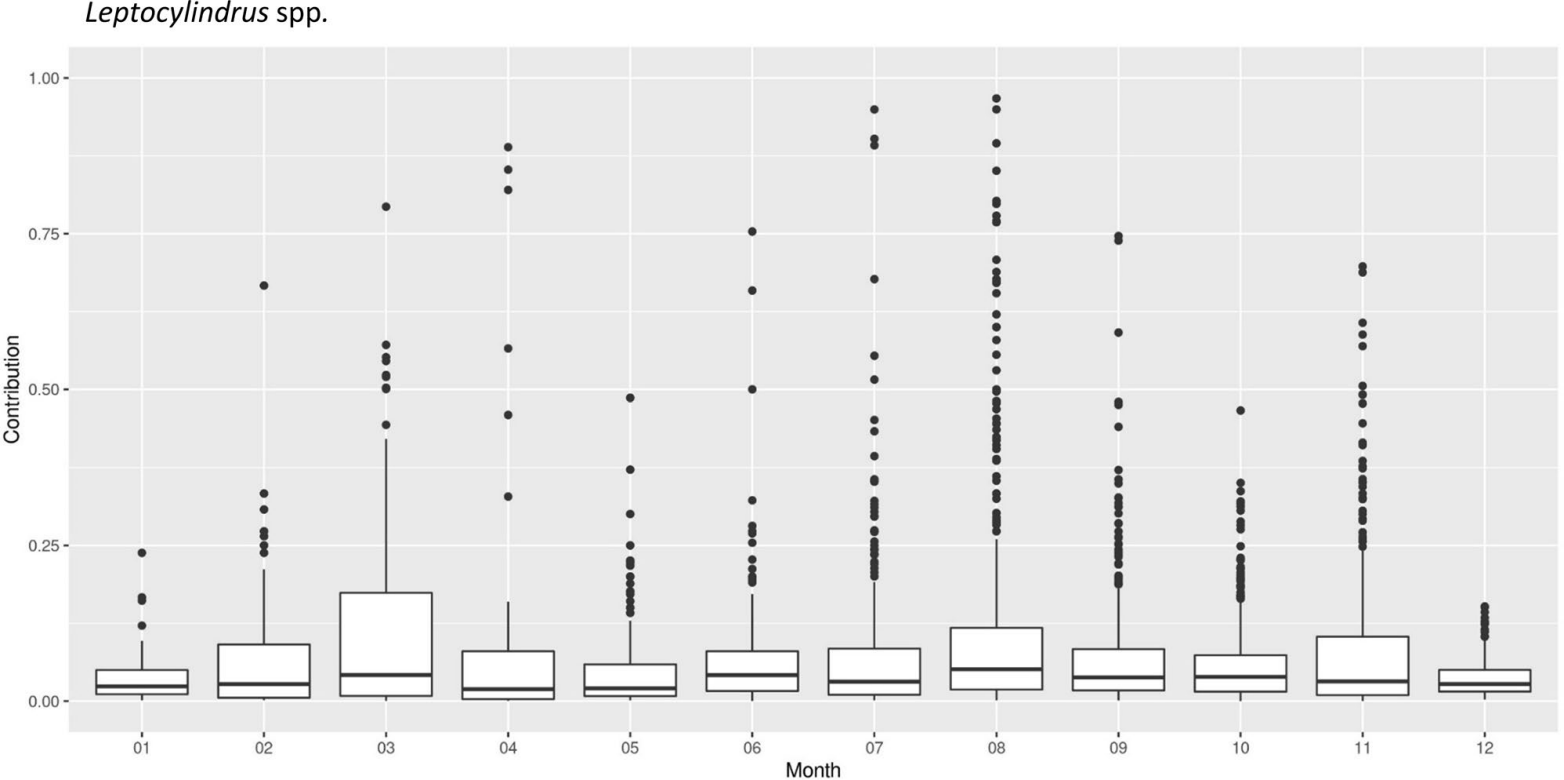
Box and whisker plot of the contributions of *Leptocylindrus* spp. throughout the year to the total microphytoplankton abundance for the years 1978–2020. Contribution is calculated as total microphytoplankton abundance divided by *Leptocylindrus* spp. Abundance.

**Figure 4:**
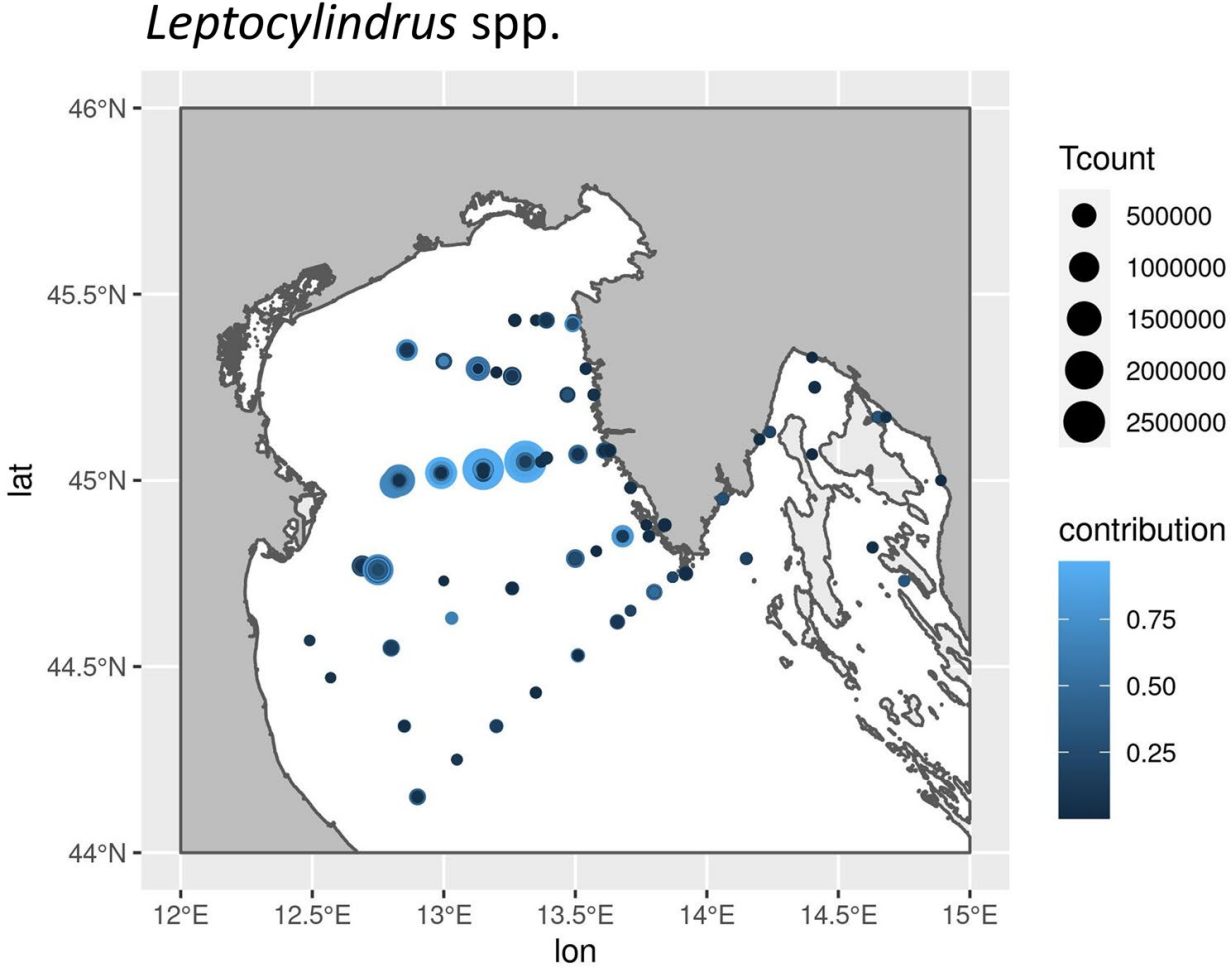
Spatial distribution of *Leptocylindrus* spp in the northern Adriatic. The area of the circles marking the sampling positions represents the maximum abundance recorded for the position. The shade indicates the contribution relative to the total microphytoplankton abundances at sampling time.

*L*. *aporus* and *L. hargravesii* are fairly new species and in our long term dataset (years 1978–2020) they are combined within *Leptocylindrus* spp. Our long-term data for *Leptocylindrus* species demonstrate regular elevated contribution [as (total microphytoplankton abundance)/(*Leptocylindrus* spp. abundance)] in March, August and November (Fig. 3). Throughout the northern Adriatic high contributions and high total count (Tcount) of *Leptocylindrus* spp. were recorded at latitude 45°N where the Po river plume reaches eastward across the northern Adriatic and the nutrient rich waters are more and more depleted of dissolved inorganic phosphate with increasing longitude (Fig. 4).

### Growth dynamics

**Figure 5:**
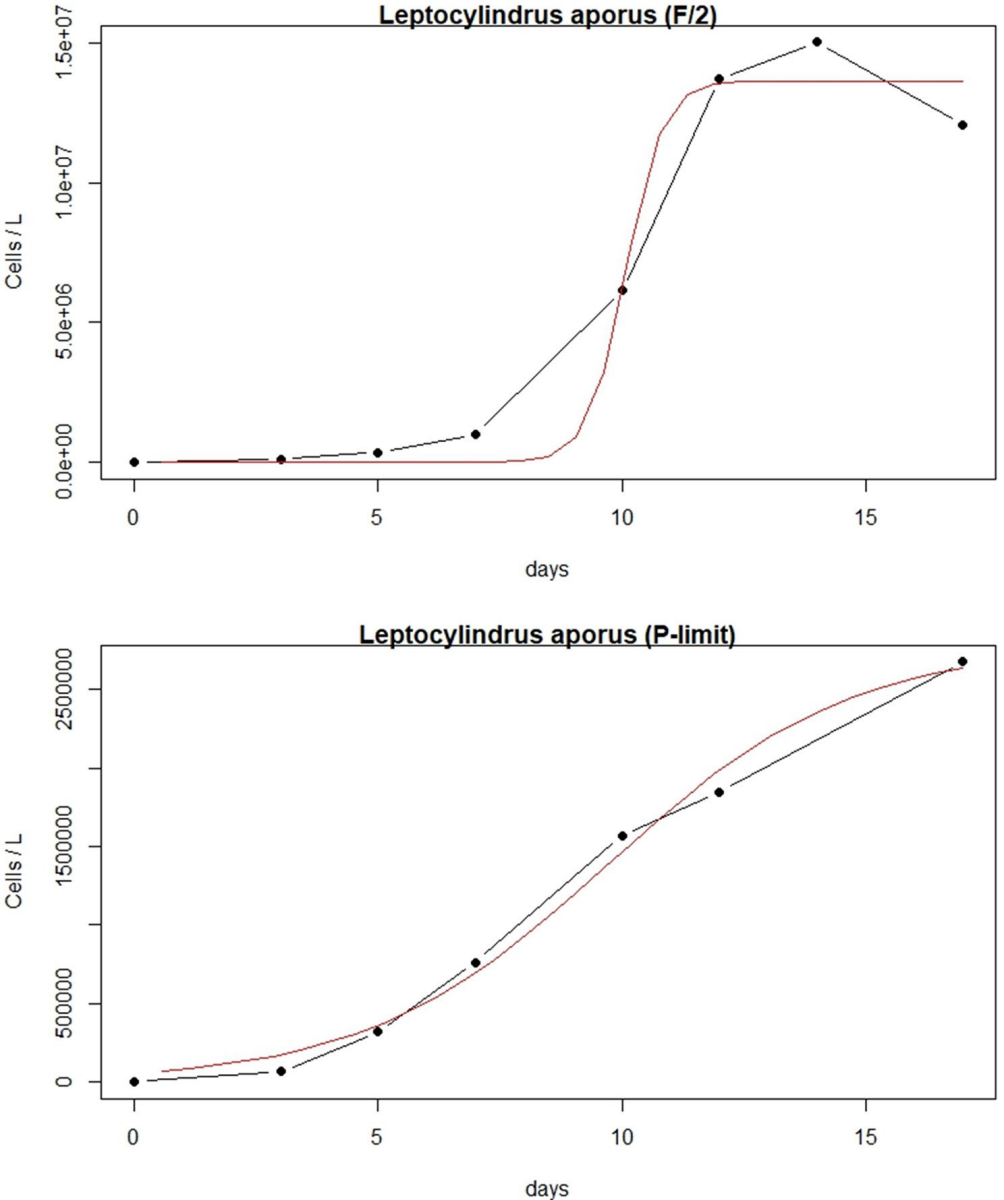
Growth curves of *L. aporus*: averaged cell concentrations in F/2 and P-limit medium throughout 17 days of experiment duration.

**Figure 6:**
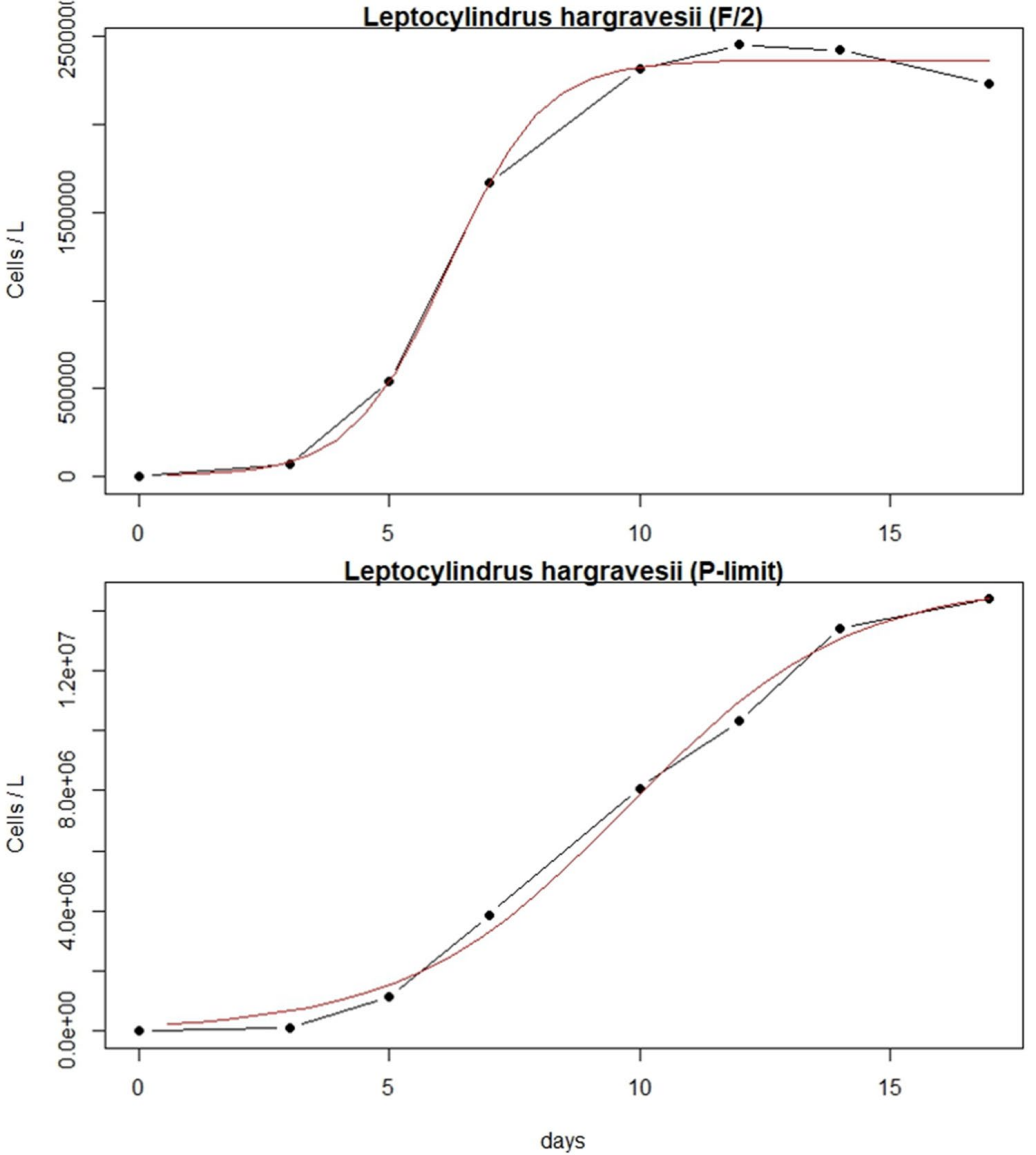
Growth curves of *L. hargravesii*: averaged cell concentrations in F/2 and P-limit medium throughout 14 days of experiment duration.

Cultures in full medium (F/2) as well as in P-limit medium (F/2 without added PO_4_^3−^) were started at cell concentrations of 82.9 × 10^3^ cells L^−1^ for *L. aporus* and 16.8 × 10^3^ cells L^−1^ for *L. hargravesii.* Both cultures in both media reached the beginning of the exponential and stationary phases on different days. *L. aporus* reached the beginning of the exponential phase after 7 days in F/2 medium and stationary phase after 12 days. In P-limit medium the same species reached the beginning of the exponential phase after 3 days and a stationary phase after 17 days (Table 1). *L. hargravesii* reached the beginning of the exponential phase after 3 days in both medium and stationary phase on different days, in F/2 after 10 days and in P-limit medium after 14 days (Table 1). *L. aporus* in F/2 medium reached the inflection point (shortest generation time) on average after 10.1 days, while the cultures in P-limit reached that inflection point on average after 9.72 days. Shortest generation time for cultures in F/2 was on average 0.26 days, while for those in P-limit it was 1.72 days (Fig. 5, Table 1)*. L. hargravesii* cultures in F/2 reached the inflection point on average after 6.16 days, while the cultures in P-limit reached that inflection point on average after 9.77 days. Shortest generation time for cultures in F/2 was on average 0.66 days, while tor those in P-limit it was 1.53 days (Fig. 6, Table 1).

**Table 1:** Growth dynamics of *L. aporus* and *L. hargravesii* shown by cells/L on various days.

Species	Medium	Beginning of exponentional growth			Inflection point (shortest cell division times)			Beginning of stationary phase		
		(days)	(cells/L)	stdev	(days)	(cells/L)	stdev	(days)	(cells/L)	stdev
*L. aporus*	F/2	7	1.07E+06	8.22E+05	10.01	6.23E+06	4.72E+06	12	1.38E+07	8.98E+06
	P-limit	3	1.48E+05	5.43E+04	9.72	1.65E+06	7.68E+05	17	2.76E+06	6.21E+05
*L. hargravesii*	F/2	3	8.63E+04	2.81E+04	6.16	1.69E+06	7.78E+04	10	2.34E+06	2.41E+05
	P-limit	3	1.08E+05	1.61E+04	9.77	8.09E+06	1.56E+06	14	1.34E+07	6.18E+05

### Morphological reactions to different nutrient conditions

**Figure 7:**
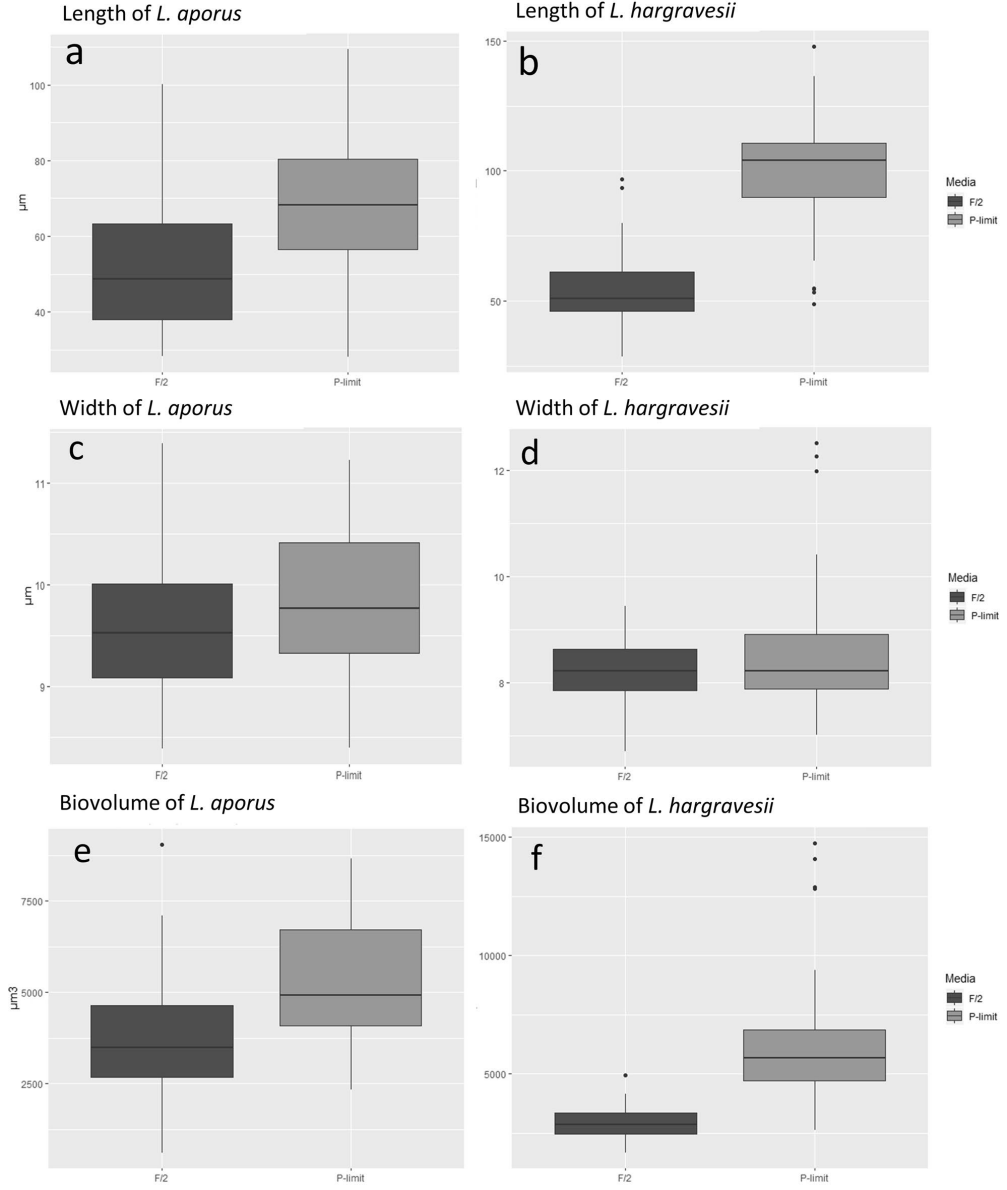
Morphology measurements. Box and whisker plots for the cell length (pervalvar axis) of *L. aporus* (**a**) and *L. hargravesii* (**b**), cell widths (apical axis) of *L. aporus* (**c**) and *L. hargravesii* (**d**). Biovolume of *L. aporus* (**e**) and *L. hargravesii* (**f**), grown in F/2 (dark grey) and P-limit (light grey) medium.

**Figure 8:**
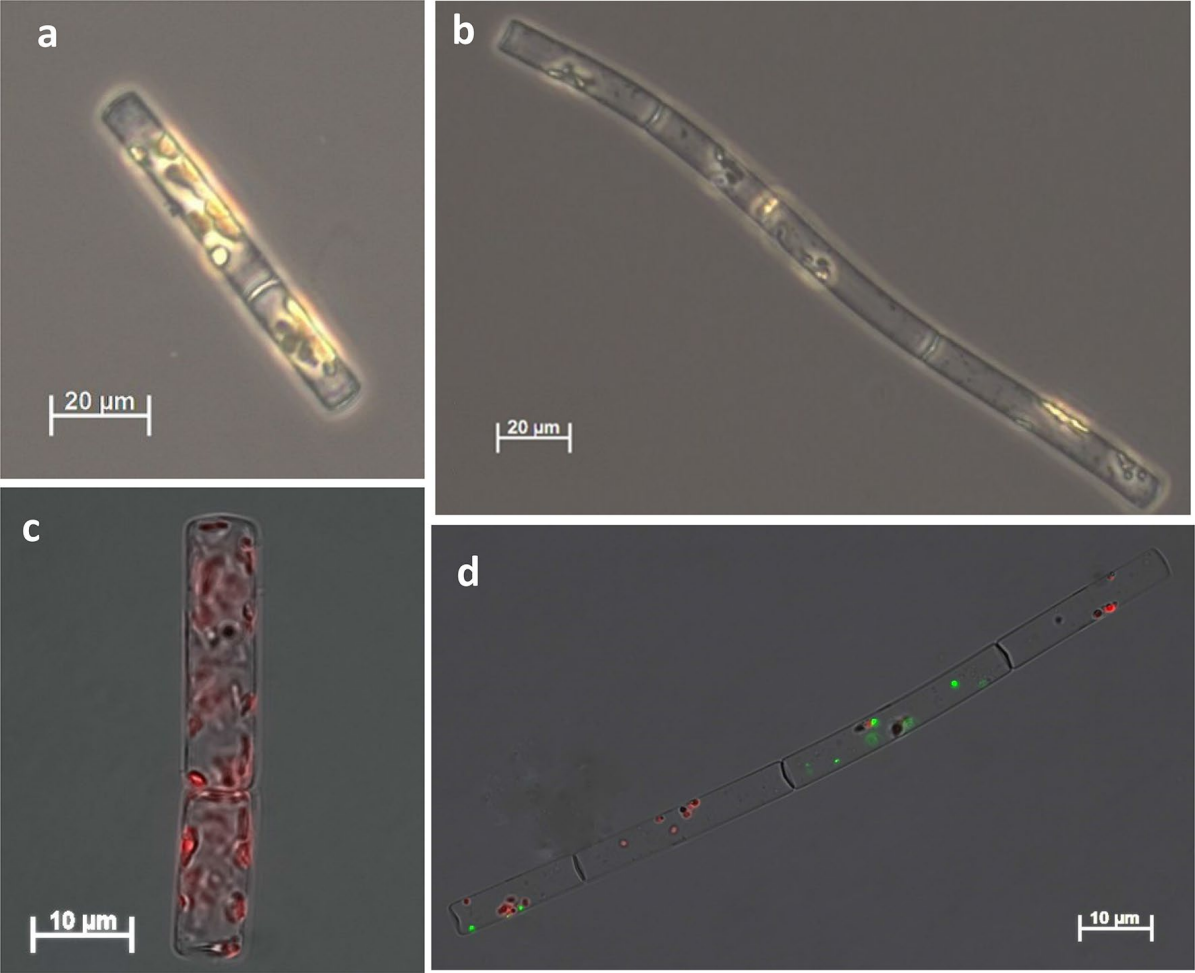
Light micrograpy of *L. aporus* (**a**–**d**). Cells of *L. aporus* grown in F/2 (**a**, **c**) and P-limit (**b**, **d**) medium. *L. aporus* cell from a culture in P-limit medium after incubation with alkaline phosphatase substrate from the ELF 97 Phosphatase Detection Kit. Chloroplast autofluorescence is shown in red. The fluorescent signal from dephosphorylated alkaline phosphatase substrate is shown in green. It accumulates at the location of alkaline phosphatase activity, along the cell.

**Figure 9:**
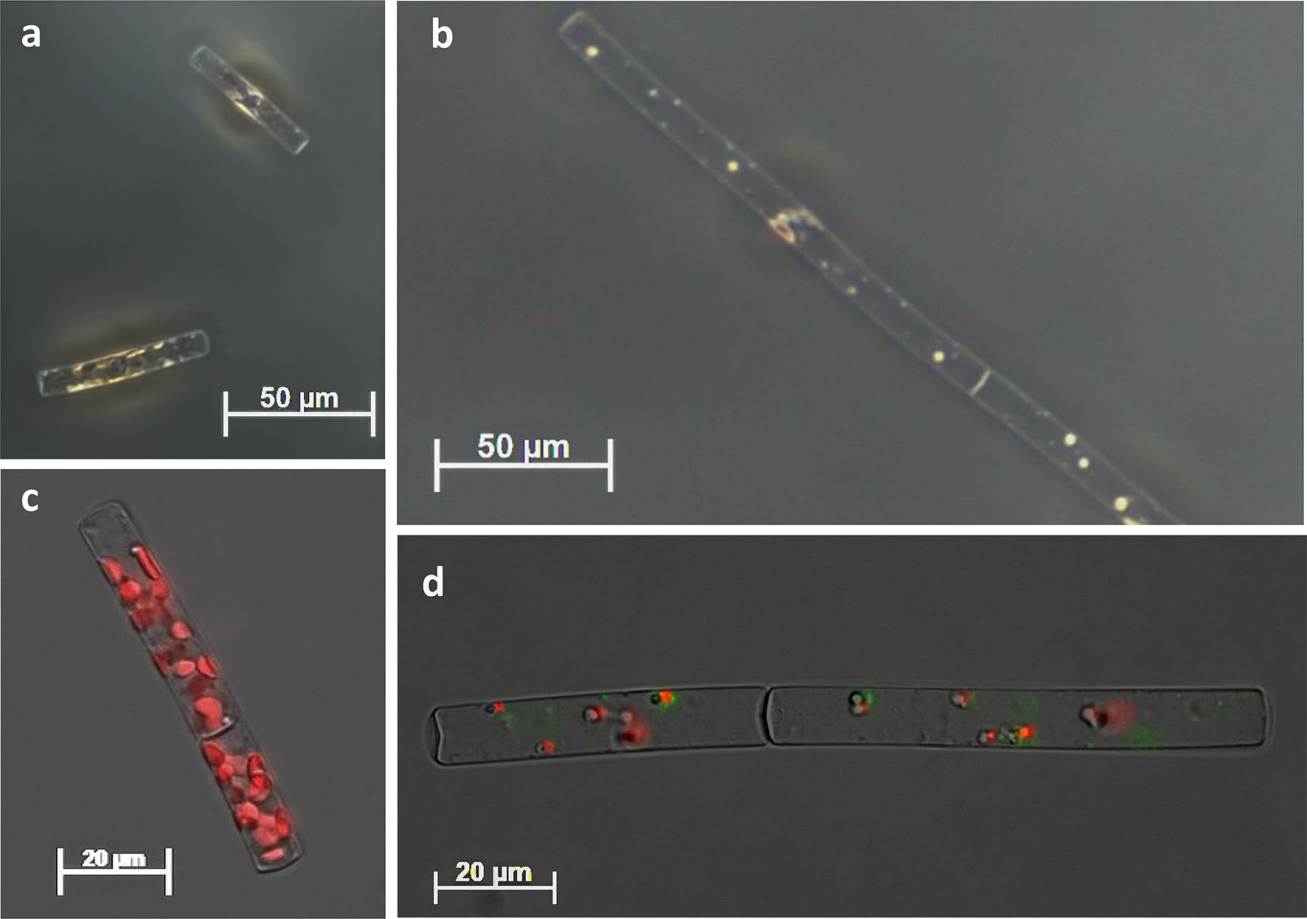
Light micrograpy of cells of *L. hargravesii* (**a**, **b**) grown in F/2 (**a**, **c**) and P-limit (**b**, **d**) medium. *L. hargravesii* cell from a culture in P-limit medium after incubation with alkaline phosphatase substrate from the ELF 97 Phosphatase Detection Kit. Chloroplast autofluorescence is shown in red. The fluorescent signal from dephosphorylated alkaline phosphatase substrate is shown in green. It accumulates at the location of alkaline phosphatase activity, along the cell.

Both *L. aporus* and *L. hargravesii* cells grown in P-limit medium showed significant morphological differences if compared to cells grown in F/2 medium. Number of analyzed cells in F/2 and P-limit medium for *L. aporus* was 30 and 45 and for *L. hargravesii* 27 and 50 cells respectively. Significant differences for cell lengths (pervalvar axis) of both *Leptocylindrus* species (*p* value_(*L. aporus*)_ = 3.13 × 10^–4^ and *p* value_(*L. hargravesii*)_ = 4.42 × 10^–14^) were revealed with Welch two sample *t* test (Fig. 7, Table 2). Average cell length for *L. aporus* was 53.05 μm in F/2 and 69.86 μm in P-limit medium and for *L. hargravesii* was 54.92 μm and 104.82 μm for cells grown in F/2 and P-limit medium respectively. A Welch two sample *t* test also revealed that there was no significant difference of cell width between different medium for both *Leptocylindrus* species (*p* value_(*L. aporus*)_ = 6.8 × 10^–2^ and *p* value_(*L. hargravesii*)_ = 1.10 × 10^–2^) (Fig. 7, Table 2). A Welch two sample *t* test also revealed significant differences for average cellular biovolume for both species (*p* value_(*L. aporus*)_ = 5.07 × 10^–4^ and *p* value_(*L. hargravesii*)_ = 3.23 × 10^–11^). Calculated average cell biovolume was 3.82 × 10^3^ µm^3^ in F/2 and 5.29 × 10^3^ µm^3^ P-limit medium for *L. aporus* and 2.90 × 10^3^ µm^3^ and 6.19 × 10^3^ µm^3^ for *L. hargravesii* cells grown in F/2 and P-limit medium, respectively (Fig. 7, Table 2). Differences between *L. aporus* and *L. hargravesii* cells grown in two different nutrient conditions seen by light microscopy are presented in Figs. 8 and 9 respectively.

**Table 2:** Morphometric parameters of *L. aporus* and *L. hargravesii*: length and width of cells shown in µm and cell biovolume in µm^3^.

Species	Medium	Length			Width			Biovolume		
		(um)	stdev	*p*	(um)	stdev	*p*	(um)	stdev	*p*
*L. aporus*	F/2	53.05	18.53	3.13E−04	9.40	1.05	6.80E−02	3.82E+03	1.75E+03	5.07E−04
	P-limit	69.86	18.92		9.80	0.66		5.29E+03	1.58E+03	
*L. hargravesii*	F/2	54.92	15.99	4.42E−14	8.25	0.65	1.10E−01	2.90E+03	7.60E+02	3.23E−11
	P-limit	104.82	31.18		8.59	1.18		6.19E+03	2.69E+03

Analysis of average chain lengths showed that the chains of both *Leptocylindrus* species were significantly longer in P-limit medium than in F/2 during the entire experiment. Number of analyzed chains for *L. aporus* in F/2 medium on average was 764 (ranging between 205 and 1241 analyzed chains each day of the experiment) and in P-limit 435 (ranging between 189 and 687 analyzed chains each day of the experiment). For *L. hargravesii* in F/2 medium the analyzed chain on average was 595 (ranging between 44 and 1184 analyzed chains each day of the experiment) and in P-limit medium 433 (ranging between 43 and 868 analyzed chains each day of the experiment) analyzed chains.

**Figure 10:**
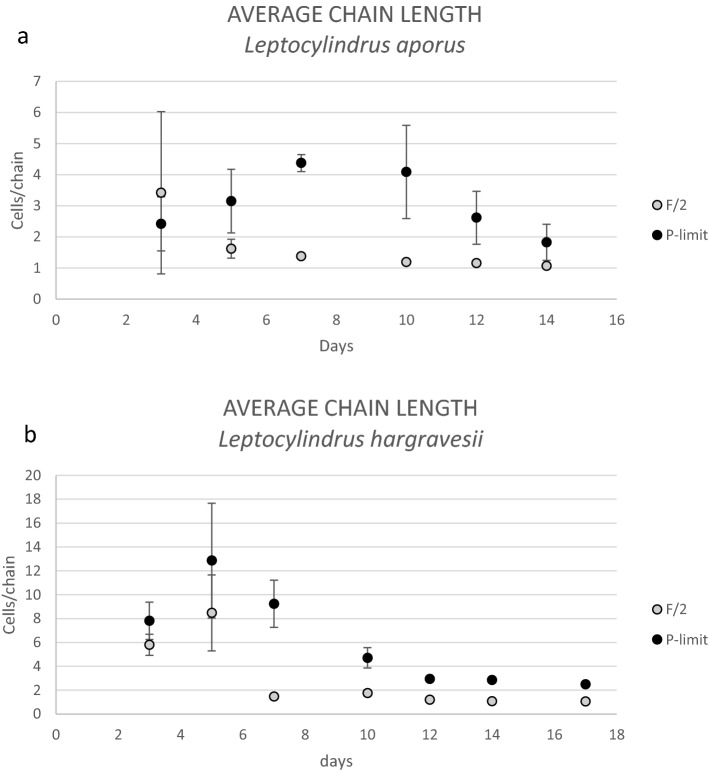
Average chain lengths in F/2 and P-limit medium for *L. aporus* (**a**) an *L. hargravesii* (**b**) during 17 days of the experiment.

*L. aporus* had a maximum average chain length of 3 cells chain^−1^ on a third day of experiment and 4 cells chain^−1^ on seventh and eighth day in F/2 or P-limit medium, respectively. Minimum average chain length was 1 cell chain^−1^ from seventh till seventeenth day in F/2 medium, while in P-limited medium minimum average chain length was 2 cells chain^−1^ on the third and fourteenth day of the experiment (Fig. 10).

*L. hargravesii* had maximum average chain length of 8 cells chain^−1^ and 13 cells chain^−1^ on the fifth day of experiment in F/2 or P-limit medium, respectively. Minimum average chain length was 1 cell chain^−1^ on seventh and twelfth till seventeenth day in F/2 medium, while in P-limited medium, minimum average chain length was 2 cells chain^−1^ on the seventeenth day (Fig. 10).

### Chlorophyll fluorescence intensity

**Figure 11:**
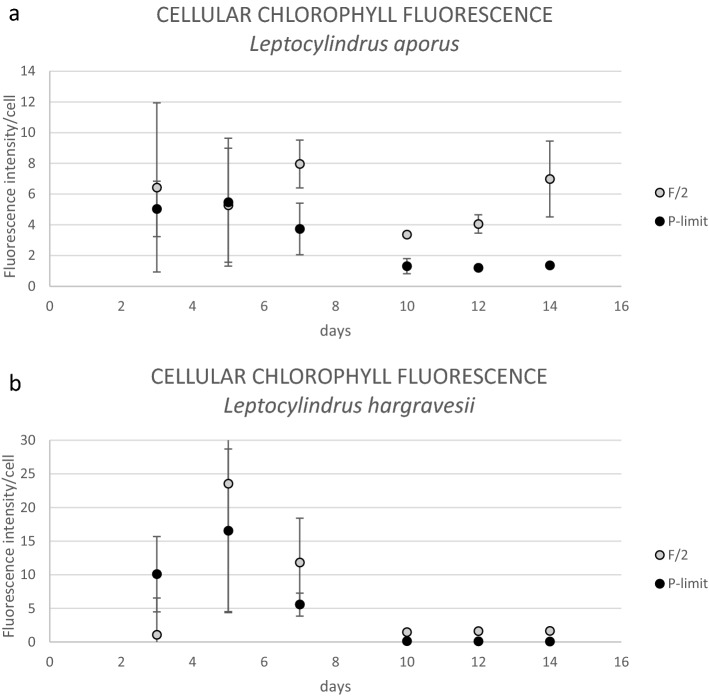
Fluorescence intensity/cell between F/2 and P-limited medium for both investigated species, *L. aporus* (**a**) and *L. hargravesii* (**b**).

Analysis of cellular chlorophyll fluorescence intensity/cell of both *Leptocylindrus* species was significantly higher in F/2 than in P-limited medium during whole experiment. The biggest difference between P-limit medium and F/2 medium was recorded on the last day of the experiment for both species. During the lag phase of both cultures, low cell numbers resulted in relatively large standard deviations (Fig. 11). Differences between *L. aporus* and *L. hargravesii* cells grown in two different nutrient conditions seen by epifluorescent microscopy are presented in Figs. 8c, d and 9c, d respectively. Chloroplasts (shown in red) as detected by their CHLa fluorescence clearly are smaller in cells grown in P-limit medium.

### Alkaline phosphatase activity (APA) and subcellular localization

**Figure 12:**
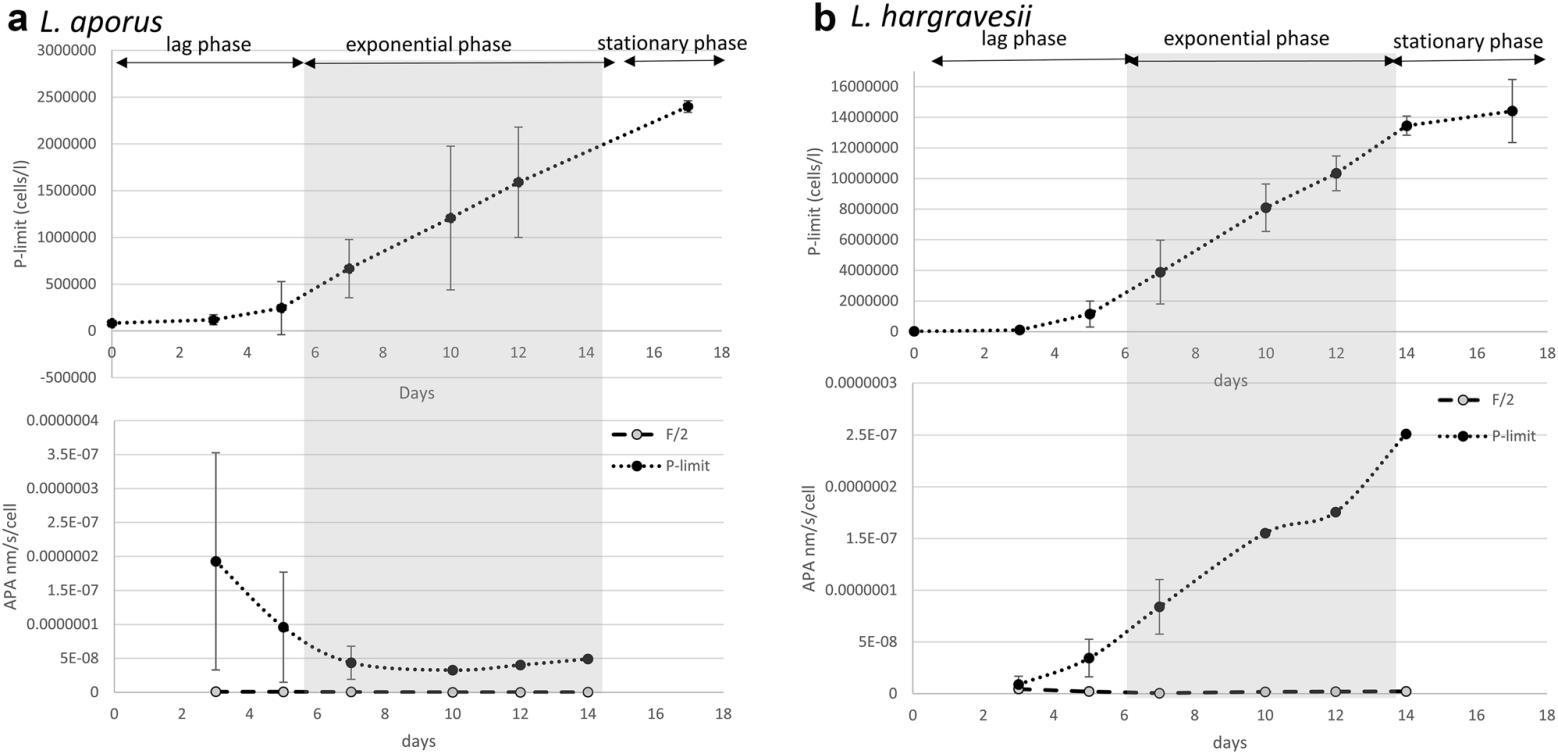
The dynamics of cellular APA of cultures in F/2 and P-limit medium across the growth curves of respective cultures in P-limit medium; (**a**) *L. aporus*, (**b**) *L. hargravesii.*

**Figure 13:**
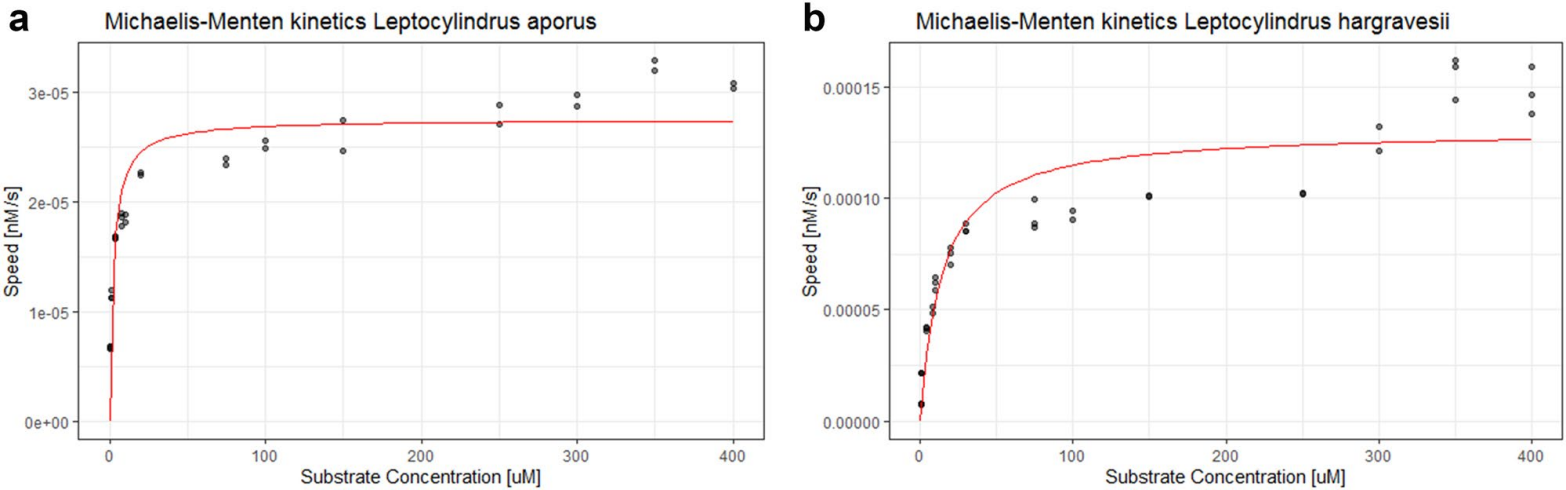
The AP enzyme kinetics of *L. aporus* (**a**) and *L. hargravesii* (**b**).

Cellular APA was calculated as measured APA divided by the number of cells in the sample. For both species APA was measured in both media (F/2 and P-limit). For *L. aporus* in P-limited medium we detected a maximal APA of 694 fmol h^−1^ cell^−1^ at the beginning of the experiment, activity decreased during lag and exponential growth phase and at the beginning of stationary phase APA again started to increase and was increasing up to 176 fmol h^−1^ cell^−1^. For *L. hargravesii* in P-limited medium we detected minimal APA at the beginning of experiment and that activity continuously increased through exponential growth phase to a maximum of activity of 904 fmol h^−1^ cell^−1^ at the end of the exponential growth phase. In F/2 medium low cellular APA was detected for both species. For *L. aporus* (Fig. 8d) was relatively high already at the first APA measurement at day 2 (3 fmol h^−1^ cell^−1^) from when activity continuously decreased from the beginning of experiment (3 fmol h^−1^ cell^−1^) until the end of the exponential growth phase, when it slowly increased again up to 0.8 fmol h^−1^ cell^−1^ (Fig. 12a). For *L. hargravesii* (Fig. 9d) a maximal activity (16 fmol h^−1^ cell^−1^) was detected at the beginning of the exponential phase, it continuously decreased to a minimum of 2 fmol h^−1^ cell^−1^ in the middle of exponential phase, then started to increase again until day 14 when it reached 8 fmol h^−1^ cell^−1^ (Fig. 12b). The analysis of the AP enzyme kinetics showed a half saturation constant (K_m_) value of 2.51 µM for *L. aporus* (Fig. 13a) and 13.9 µM for *L. hargravesii* (Fig. 13b) which indicates a high affinity enzyme for both species. After incubation of *L. aporus* and *L. hargravesii* cells with substrate for AP from the ELF 97 Endogenous Phosphatase Detection Kit, the fluorescent product accumulated on the surface of the cells. The green signal seen on both *Leptocylindrus* species (Figs. 8d, 9d) indicates the localization of APA on the cells. The fluorescent product accumulated in several small areas on the cell surface.

### The dynamics of dissolved inorganic phosphate uptake

**Figure 14:**
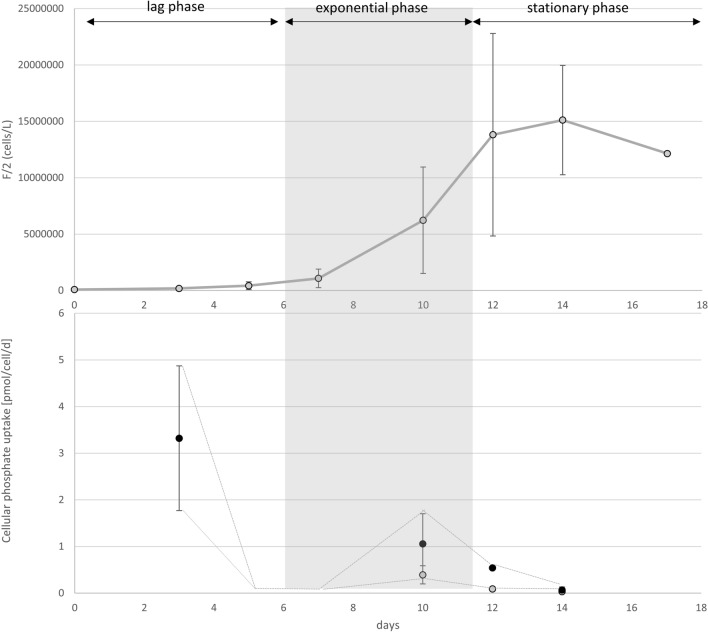
The dynamics of phosphate uptake rates for *L. aporus* throughout the experiment duration of batch cultures in F/2 medium. Dynamics of phosphate uptake rates throughout batch cultures of *L. aporus* in F/2 medium. (**a**) Average cell concentrations (and standard deviations) plotted over the duration of the batch culture in days. The phases of the growth curve are indicated. (**b**) Cellular phosphate uptake rates calculated as phosphate uptake divided by cell numbers at the days of phosphate quantification (blue, or lower values) and divided by the cell numbers at the day of the measurement before (orange, or higher values). The dashed line indicates lower and upper borders between the two calculation methods. The true uptake rate must be between the two borders. Note the steep drop of phosphate uptake rates during exponential growth.

**Figure 15:**
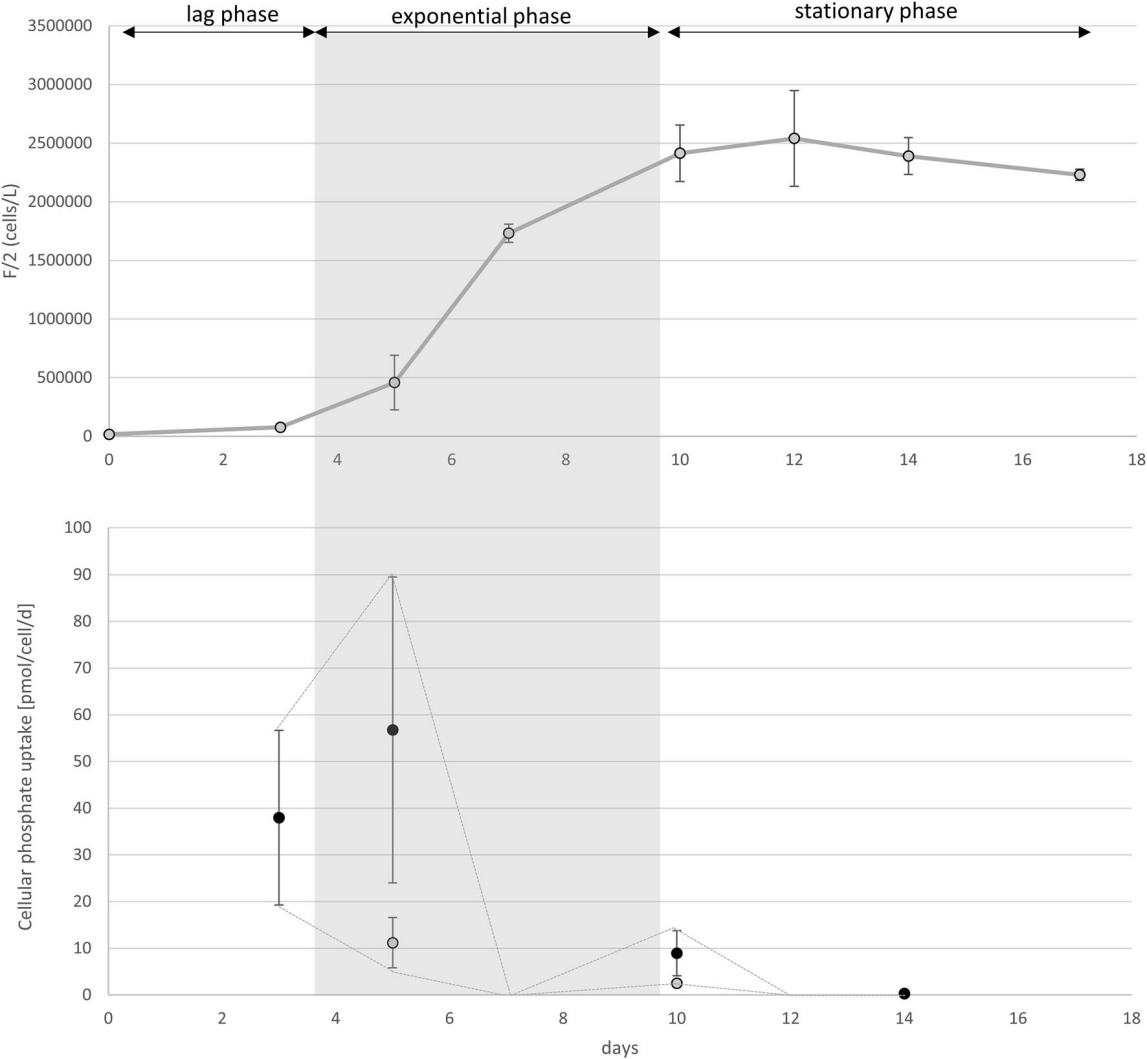
The dynamics of phosphate uptake rates for *L. hargravesii* throughout the experiment duration of batch cultures in F/2 medium. Dynamics of phosphate uptake rates throughout batch cultures of *L. hargravesii* in F/2 medium. (**a**) Average cell concentrations (and standard deviations) plotted over the duration of the batch culture.in days. The phases of the growth curve are indicated. (**b**) Cellular phosphate uptake rates calculated as phosphate uptake divided by cell numbers at the days of phosphate quantification (blue, or lower values) and divided by the cell numbers at the day of the measurement before (orange, or higher values). The dashed line indicates lower and upper borders between the two calculation methods. The true uptake rate must be between the two borders. Note the steep drop of phosphate uptake rates during exponential growth.

Calculated cellular PO_4_ uptake rates in F/2 medium for *L. aporus* were between 0.03 and 3.32 pmol d^−1^ cell^−1^ and for *L. hargravesii* were between 0.29 and 56.75 pmol d^−1^ cell^−1^. Concentrations of phosphate in P-limit cultures through the experiments were below our detection limit (0.02 μM), for which no phosphate uptake rates were calculated. The dynamics of phosphate rates for both species showed increased cellular phosphate uptake rates in the early phase of growth acceleration, however, for *L. hargravesii* we observed, that uptake rates increased and stayed high also during the early exponential growth phase. Dramatically reduced uptake rates during the exponential growth phase and a slight increase of uptake rates at the beginning of the stationary phase were observed for both species (Figs. 14, 15).

### Cellular lipid composition

General distributions of cellular lipid classes, PG, PE, PC, MGDG, DGDG, SQDG, ST, PIG, TG and SE, did not statistically differ between cultures grown in F/2 and in P-limit conditions (Table 3). The most dominant class are phospholipids, contributing almost 60% to total cellular lipid content.

**Table 3:** *L. aporus* and *L. hargravesii* cellular lipid classes distribution (%) for the F/2 and P-limit conditions.

	PG	PE	PC	MGDG	DGDG	SQDG	ST	PIG	TG	SE
	%									
***L. aporus***										
*F/2*										
%	**35.2**	**21.2**	**1.1**	**13.5**	**3.4**	**12.3**	**0.7**	**6.1**	**3.6**	**2.7**
stdev	4.1	2.1	0.6	4.8	2.2	2.4	0.2	0.7	0.5	1.9
*P-limit*										
%	**33.9**	**24.3**	**1.1**	**10.2**	**3.0**	**16.6**	**0.6**	**6.2**	**2.5**	**1.6**
stdev	1.5	2.7	0.7	1.4	2.1	3.7	0.2	1.3	1.0	0.5
P *t* test	0.33	0.16	0.50	0.23	0.28	*0.02*	0.16	0.49	0.09	0.18
***L. hargavesi***										
*F/2*										
%	**34.3**	**22.9**	**0.7**	**8.4**	**3.3**	**22.0**	**0.5**	**3.5**	**3.5**	**0.9**
stdev	1.7	5.7	0.5	3.2	1.4	5.4	0.3	1.1	2.2	0.5
*P-limit*										
%	**35.3**	**19.7**	**1.1**	**14.0**	**4.6**	**13.3**	**1.3**	**5.0**	**5.0**	**0.8**
stdev	0.1	1.3	0.5	0.8	0.9	3.1	0.1	0.4	0.8	0.3
P *t* test	0.21	0.18	0.29	0.07	0.16	0.06	*0.01*	0.09	0.10	0.36

In general, there were no significant differences (*P* > 0.05) between the cells grown in F/2 and in P-limit medium. Exceptions were SQDG for *L. aporus* and ST for *L. hargravesii.*

Significance values of P* t* test are in bold and italics.

## Discussion

### Growth dynamics

To understand how two *Leptocylindrus* species are reacting to conditions with high and low phosphate availability, we observed batch cultures grown in F/2 (balanced overabundance of all nutrients) and P-limit (F/2 medium without phosphates) medium. *L. aporus* and *L. hargravesii* showed significant differences in growth dynamics between the two conditions. *L. aporus* cells grown in P depleted conditions started their exponential growth slightly (1 day) earlier than those from F/2 medium. This indicates a slight preference for P-depleted conditions, which might indicate that *L. aporus* competes better for organic phosphorus in P-depleted conditions. But at the start of the stationary phase, the cultures reached lower cell concentrations in P depleted medium than those grown in P replete conditions (Fig. 5). This species reacted on P depleted conditions with an early onset of accelerated cell division but also initiated stationary phase at lower cell concentrations in P depleted than in P replete medium. This suggests P limitation at the end of the batch culture (limitation/inhibition due to high cell concentrations is less likely when we take maximal concentrations from the P replete medium into account). In situ, organic P sources if available can be used to sustain the bloom. Similar reactions to P limitation were also reported for other diatom species such as *Phaeodactylum tricornutum* or *Chaetoceros peruvianus*^12,26^. *L. hargravesii* cells on the other hand in both media initiated exponential growth phase on the same day of their respective batch cultures. In the P depleted conditions this phase lasted longer and cells reached much higher cell concentrations than in P replete conditions (Fig. 6). This species seems to grow faster and to higher abundances in conditions when inorganic P is not abundantly available. Comparable observations have been reported by Finenko et al.^22^. For *L. hargravesii* we have to assume that the species seeks conditions with very limited or no availability of dissolved inorganic phosphates to outcompete other plankton species and form a bloom.

### Morphological reactions to different phosphorus conditions

Both *Leptocylindrus* species showed similar and significant morphological differences between the cells grown in F/2 and P-limit medium. The main morphological alteration of cells grown in P depleted conditions is a significantly elongated pervalvar axis if compared to P rich conditions. Width measurements of the *L. aporus* cells fall within the originally described ranges by Nanjappa et al., while lengths of the pervalvar axis of cells in F/2 and P-limit medium were longer than lenghts described by Nanjappa et al.^1^ with cells longer in P-limit than those in F/2 medium (Figs. 7a, 8). Morphometric characteristics of *L. hargravesii* cells grown in F/2 medium fall within the originally described ranges by Nanjappa et al.^1^, but those in P-limit medium are almost twice longer (Figs. 7b, 9). Due to elongation of pervalvar axis the overall cell biovolume is higher for both species. For the diatom species *Chaetoceros peruvianus* we could recently describe not only the elongation of the pervalvar axis, but also the significant elongation of setae, which in the case of *C. peruvianus* is the localization of all APA. *C. peruvianus* hence specifically increases cell surface area^12^ where APA is located, but also increases cell volume by elongation of the pervalvar axis. The here investigated *Leptocylindrus* species in a very similar manner increase their cell volume by elongation of the pervalvar axis, and at the same time increase the surface area of the part of the cell where APA is located. This elongation of the cells is achieved by increasing the number of girdle bands. Another morphological alteration of cells grown in P-depleted conditions is the increase in average chain length during the whole experimental period for both *Leptocylindrus* species (Fig. 10). Forming longer chains might be a result of more stable links between the cells. In any case longer chains are most likely rendering the species more resilient to predation as is shown for e.g. *Skeletonema marinoi*^52^. This observation hence is another adaptation of the two *Leptocylindrus* species to better compete in phosphate limited conditions. The aforementioned morphological changes that are induced by phosphate limitation can also be considered a helpful tool in ecological observations. For the two species observed here, the described morphological changes can be used to derive insights into ecological conditions by morphological analysis of diatom samples. This might find applications in sediment/fossil climatology as well as in the analysis of recent and historical phytoplankton samples in climate change research.

### Influence of phosphorus availability on chlorophyll content

Chlorophyll fluorescence intensity for both *Leptocylindrus* species showed significant differences between the two observed growth conditions. During the whole experiment chlorophyll fluorescence intensity was lower in the cells grown in P-limit medium for both *Leptocylindrus* species, so these species have the same response as many other species previously described like *Pseudo-nitzschia multiseries*^24^, *Chaetoceros glacialis*^23^, *Chaetoceros curvisetus, Skeletonema costatum*^22^, *P. tricornutum*^26^ and *Thalassiosira weissflogii*^25^. In those experiments it is demonstrated that the various processes that take place in cells under phosphorus limitation also affect the synthesis of pigments. It is hypothesized that with a lack of phosphorous, pigment synthesis is stopped because cells are no longer able to synthesize enough RNA. During phosphorus limitation, the concentration of CHLa per cell often decreases. This is yet to be investigated in detail for *Leptocylindrus* species. However, our results demonstrate, that for both investigates species, cells grown in phosphorous depleted conditions show less CHLa fluorescence, and fewer and smaller chloroplasts. The decrease in chloroplast size and number as well as the decrease in CHLa fluorescence might be considered and adverse effect of phosphorous depletion. Since we noticed higher contributions to the phytoplankton community as well as higher abundances for both species from March to October, when light intensity in the northern Adriatic is generally high, we may suggest that the two investigates species can generate a competitive advantage even with reduced photosynthetic capacity.

### Adaptation to phosphorus deficiency by alkaline phosphatase induction

Cellular APA for *L. aporus* had a maximum at the beginning of the experiment with 694 fmol h^−1^ cell^−1^. During the lag and exponential phase APA continually decreased until the beginning of stationary phase when it started to slowly increase and at the end of experiment reached 176 fmol h^−1^ cell^−1^. Phosphorus limited *L. aporus* cells accumulated maximal amounts of AP during the lag phase and slightly increased at the end of the exponential growth phase (Fig. 12a). This observation might be explained by reduced S phase (cell cycle phase) duration during exponential growth and reduced expression of alkaline phosphatases during phases of elevated growth rates.

A fast APA increase as soon as phosphorous depletion is detected helps the species to compete for organic phosphorous resources just before it’s in situ bloom phase, and the increase at the end of the exponential growth phase helps the species to compete for organic phosphate at the peak of its bloom in situ. AP enzyme kinetics (Fig. 13a) showed that this species’ exhibits a relatively low K_m_ value of 2.51 µM which indicates high specificity for substrate and indicates high ability to compete for organic phosphate when substrate concentrations are low.

We observed different APA dynamics for *L. hargravesii* for which APA increased during the entire experiment (Fig. 12b). This implies, that despite increased cell division rates *L. hargravesii* was able to increase cellular APA (and presumably AP density on the cell surface) throughout the exponential growth phase. This means that *L. hargravesii *in situ can make use of organic phosphate resources with increasing efficiency during its bloom establishment. AP enzyme kinetics (Fig. 13b) showed that this species has a relatively high K_m_ value of 13.9 µM which indicates lower specificity for substrate and an adaptation to higher concentrations of organic phosphate. Blooms of this species can be hence expected under conditions where dissolved inorganic phosphate is depleted, but organic phosphate resources are available at relatively high concentrations. In comparison to e.g. *Chaetoceros peruvianus* (APA K_m_ value of 64.59 μM) the APA K_m_ value of *L. hargravesii* still is not on the lower side of the scale^12^. Accordingly, *L. hargravesii* reaches much higher relative abundances and total cell counts than *C. peruvianus* in the northern Adriatic where competition for phosphorous resources is one of the driving characteristics of it’s pelagic ecosystem^4^*.* This notion is corroborated by in situ observations (Figs. 2, 3). Figures 2 and 3 show that the here investigated species regularly dominate microphytoplankton communities in early spring and late summer and with increasing dominance from the PO river mouth towards the eastern coast of the northern Adriatic Sea. Early spring is characterized by elevated freshwater input to the area which results in elevated but phosphorous depleted nutrient conditions, conditions that *L. aporus* appears to be well adapted to. Later in the year, in late summer and early winter, we often found conditions of nutrient exhaustion, phosphorus depletion and elevated concentrations of organic phosphates as a result of bloom termination and higher grazing. Furthermore, the planktonic community uses up dissolved inorganic nutrients while drifting eastwards away from the river PO mouth towards the eastern coast of the northern Adriatic, which again favors species adapted to competition for organic P towards the eastern coast. On the way organic nutrient concentrations increase or are increasingly important as nutrient sources, thus favoring species that are adapted to utilize organic nutrients like e.g. organic phosphates. *L. hargravesii* appears to be well adapted to those conditions and might well be responsible for the *Leptocylindrus* late summer and autumn blooms observed.

To determine cell AP location, we used ELF 97 marking. The fluorescent non soluble product accumulated on the cell surface of both species (Figs. 8, 9) and showed where APA is located. We detected AP only on the cells grown in P-limited conditions, which is one more indicator that these species synthesize AP only in P-limited conditions. *Leptocylindrus* cells specifically enlarged cell surface areas where APA is localized when deprived of inorganic phosphates, as observed for other diatom species such as *C. peruvianus*^12^.We found that APA was localized where the girdle bands build the cylindrical shape of the cells, the part of the cell surface that dramatically increased when cells grew in phosphorous depleted conditions (Fig. 7).

### The dynamics of dissolved inorganic phosphate uptake

For both *Leptocylindrus* species we detected similar behavior for cellular phosphate uptake in P replete conditions. We detected high and increasing phosphate uptake rates during the lag phase, followed by a steep decrease at the end of lag phase till the middle of exponential phase. *L. hargravesii* maintained higher uptake rates for longer period of the exponential growth phase. At the end of exponential phase and the start of stationary phase cellular phosphate uptake slightly increased and during stationary phase continued to decrease (Figs. 14, 15). It is fair to assume that the strategy of both *Leptocylindrus* species is to load up phosphate when limitation is sensed and then perform a bloom on the accumulated P-pools. It has been experimentally proven that this is the case also for some other species such as *Thalassiosira pseudonana* that stores polyphosphates when is found in P stress conditions^53^.

### Cellular lipid composition

Confronted with unfavorable environmental conditions phytoplankton reacts with physiological acclimation/genetic adaptation. Among other adaptation mechanisms they often remodel lipids. When found in P deplete conditions, cells have developed the possibility of replacing phospholipids with non-phospholipids^19–21,54^. One example of this adaptation is the species *Thalassiosira pseudonana* that under P limited conditions replaces PC by the nitrogen-containing betaine lipid diacylglyceryl-carboxyhydroxymethylcholine (DGCC) and PG by SQDG^55^. *Phaeodactilum tricornutum* also adapts to adverse conditions by replacing phospholipids with non-phospholipids, as evidenced by experimental results showing that during P limitation all phospholipids, including PC and PG, were below the detection level, while an increase was recorded of non-phospholipids synthesized on intracellular membranes [fivefold increase in diacylglyceryl-hydroxymethyl-N, N, N-trimethyl-b-alanine (DGTA)] and in plastids (doubled the amount of digalactosyldiacylglycerol (DGlaQ for one and a half)^56^. These changes significantly affect protein biosynthesis and the level of phosphorylated metabolites that can affect a number of metabolic functions, including growth and the ability to photosynthesize.

High proportion of phospholipids and relatively constant composition of *Leptocylindrus* species lipids cultured in nutrient replete and P depleted conditions suggest their evolutionary adaptation to P scarcity. They apparently developed mechanisms by which they take organic P from the environment, as confirmed by AP analysis.

## Conclusion

*L. aporus* and *L. hargravesii* are recently described species with only ultrastructural morphological differences, so there is not much in-depth information about these species and their ecology. Both species are frequently observed with high abundances and high contributions to the microphytoplankton community in the northern Adriatic Sea. We report morphological and physiological reactions of both species to phosphate deprivation, a major ecological driver in the northern Adriatic Sea, and discuss the adaptations in an ecological context.

Both *Leptocylindrus* species showed similar morphological changes in P deplete conditions. Pervalvar cellular axis and average chain lengths increase when inorganic phosphate is not available, which at the same time increases cellular surface areas available for AP.

CHLa fluorescence of both species significantly decreased in P depleted medium. How the species cope with the resulting reduced photosynthesis remains to be investigated.

*L. aporus* reacts on a pulse of inorganic phosphate with a fast increase of phosphate uptake rates, presumably filling up intracellular P pools. However, the phosphate uptake ends rather quickly before the exponential growth rate is initiated. P depletion then quickly initiates exponential growth as well as an increase of APA.

In situ this would translate to an early bloom initiation upon P depletion. APA then quickly reduces with exponential growth, indicating that a bloom would not rely on organic phosphate resources, as the observed reduction does not give the species any advantage in the competition for organic phosphate.

Elongated chains in P depleted conditions renders the species more resistant to grazing during bloom formation. At the end of exponential growth, the elongated cells increase APA again on increased cell surface areas AP. Increased APA with relatively low K_m_ value helps to sustain elevated cell numbers after the bloom in competition for low concentrations of organic phosphorous. Conditions favoring this combination of capabilities are found in the northern Adriatic early in the year, when highly imbalanced nutrient inputs result in a quick biogenic depletion of inorganic phosphates and consequently very low organic phosphate concentrations.

*L. hargravesii* (like *L. aporus*) reacts on a pulse of inorganic phosphate with a fast increase of phosphate uptake rates, presumably filling up intracellular P pools. However, (unlike *L. aporus*) the early phase of exponential growth still maintains high phosphate uptake rates, which indicates that this species might rely on the availability of inorganic phosphate resources to sustain the initiation and early phases of its blooms in situ. APA then increases throughout the entire exponential growth phase. This indicates that *L. hargravesii* during its bloom formation competes for and uses organic phosphate resources to sustain its exponential growth^57^. Elongated chains in P depleted conditions render the species more resistant to grazing during bloom formation. At the same time this species increases cell surface area available for AP. Increased APA with relatively high K_m_ value helps to sustain the elevated cell numbers after the bloom in the competition for high concentrations of organic phosphorous resources. Conditions favoring this combination of capabilities are found in the northern Adriatic later in the year, when highly imbalanced nutrient inputs (by way of freshwater input) result in a quick biogenic depletion of inorganic P however with elevated concentrations of organic phosphates as a result of e.g. biomass turnover during summer.

The spatio-temporal distribution of high abundances and contribution to microphytoplankton of *Leptocylindrus spp*. observed in the northern Adriatic Sea hence fits and corroborates the abovementioned ecological interpretation of the observed physiological reactions of *L. aporus* and *L. hargravesii *in vitro.

*L. aporus* an *L. hargravesii* are two closely related, and morphologically very similar species. Our in vitro experiments demonstrated marked differences in physiological reactions to P depletion. These observations help us to understand the sympatric existence, or even evolution, of closely related species in a virtually homogenous, planktonic environment. They might also help us to understand the hidden structure and diversity in pelagic marine environments. Using observations of physiological capabilities of phytoplankton species to understand the ecological niches they occupy will help us to understand the structures of pelagic ecosystems. These observations also help us to argue a competitive exclusion principle when discussing the paradox of plankton^58,59^.

## Supplementary Information

Supplementary Information.

## References

[1] Nanjappa D, Kooistra WH, Zingone A (2013). A reappraisal of the genus Leptocylindrus (B acillariophyta), with the addition of three species and the erection of Tenuicylindrus gen. nov. J. Phycol..

[2] Hasle, G. & Syvertsen, E. (Academic Press, 1997).

[3] Gómez F, Simão TL, Utz LR, Lopes RM (2016). The nature of the diatom *Leptocylindrus mediterraneus* (Bacillariophyceae), host of the enigmatic symbiosis with the stramenopile *Solenicola setigera*. Phycologia.

[4] Ivančić I (2012). Survival mechanisms of phytoplankton in conditions of stratification-induced deprivation of orthophosphate: Northern Adriatic case study. Limnol. Oceanogr..

[5] Ivančić I (2016). Alkaline phosphatase activity related to phosphorus stress of microphytoplankton in different trophic conditions. Prog. Oceanogr..

[6] Smodlaka N (1986). Primary production of the organic matter as an indicator of the eutrophication in the northern Adriatic sea. Sci. Total Environ..

[7] Degobbis D, Gilmartin M (1990). Nitrogen, phosphorus, and biogenic silicon budgets for the northern Adriatic Sea. Oceanol. Acta.

[8] Zavatarelli M, Raicich F, Bregant D, Russo A, Artegiani A (1998). Climatological biogeochemical characteristics of the Adriatic Sea. J. Mar. Syst..

[9] Socal G (2008). Hydrological and biogeochemical features of the Northern Adriatic Sea in the period 2003–2006. Mar. Ecol..

[10] Giani M (2012). Recent changes in the marine ecosystems of the northern Adriatic Sea. Estuar. Coast. Shelf Sci..

[11] Marić D (2012). Phytoplankton response to climatic and anthropogenic influences in the north-eastern Adriatic during the last four decades. Estuar. Coast. Shelf Sci..

[12] Smodlaka Tanković M (2018). Insights into the life strategy of the common marine diatom *Chaetoceros peruvianus* Brightwell. PLoS ONE.

[13] Marić Pfannkuchen D (2018). The ecology of one cosmopolitan, one newly introduced and one occasionally advected species from the genus Skeletonema in a highly structured ecosystem, the northern Adriatic. Microb. Ecol..

[14] Benitez-Nelson CR (2000). The biogeochemical cycling of phosphorus in marine systems. Earth Sci. Rev..

[15] Paytan A, McLaughlin K (2007). The oceanic phosphorus cycle. Chem. Rev..

[16] Price, N. M. & Morel, F. M. Role of extracellular enzymatic reactions in natural waters. (1990).

[17] Hoppe H-G (2003). Phosphatase activity in the sea. Hydrobiologia.

[18] Fields MW (2014). Sources and resources: Importance of nutrients, resource allocation, and ecology in microalgal cultivation for lipid accumulation. Appl. Microbiol. Biotechnol..

[19] Van Mooy BAS (2009). Phytoplankton in the ocean use non-phosphorus lipids in response to phosphorus scarcity. Nature.

[20] Gašparović B (2013). Adaptation of marine plankton to environmental stress by glycolipid accumulation. Mar. Environ. Res..

[21] Gašparović B (2014). Factors influencing particulate lipid production in the East Atlantic Ocean. Deep Sea Res. Part 1 Oceanogr. Res. Pap..

[22] Finenko Z, Krupatkina-Akinina D (1974). Effect of inorganic phosphorus on the growth rate of diatoms. Mar. Biol..

[23] Lombardi A, Wangersky P (1991). Influence of phosphorus and silicon on lipid class production by the marine diatom *Chaetoceros gracilis* grown in turbidostat cage cultures. Mar. Ecol. Prog. Ser. Oldendorf.

[24] Pan Y, Subba Rao DV, Mann KH (1996). Changes in domoic acid production and cellular chemical composition of the toxigenic diatom *Pseudo-nitzschia miltiseries* under phosphate limitation. J. Phycol..

[25] Liu S, Guo Z, Li T, Huang H, Lin S (2011). Photosynthetic efficiency, cell volume, and elemental stoichiometric ratios in *Thalassirosira weissflogii* under phosphorus limitation. Chin. J. Oceanol. Limnol..

[26] Alipanah L (2018). Molecular adaptations to phosphorus deprivation and comparison with nitrogen deprivation responses in the diatom *Phaeodactylum tricornutum*. PLoS ONE.

[27] Guillard, R. R. L. in *Culture of Marine Invertebrate Animals* (eds W.L. Smith & M.H. Chanley) 29–60 (Plenum Press, New York, USA, 1975).

[28] Utermöhl H (1958). Zur Vervollkommnung der quantitativen Phytoplankton-Methodik. Mitteilungen des Internationale Vereinigung für theoretische und angewandte Limnologie.

[29] Keller MD, Bellows WK, Guillard RRL (1988). Microwave treatment for sterilization of phytoplankton culture media. J. Exp. Mar. Biol. Ecol..

[30] Gračan R, Mladineo I, Kučinić M, Lazar B, Lacković G (2012). Gastrointestinal helminth community of loggerhead sea turtle *Caretta caretta* in the Adriatic Sea. Dis. Aquat. Org..

[31] Anonymous X (1975). Proposals for a standardization of diatom terminology and diagnoses. Nova Hedwig. Beih..

[32] Ross, R. *et al.* An amended terminology for the siliceous components of the diatom cell. (1979).

[33] Hillebrand H, Dürselen CD, Kirschtel D, Pollingher U, Zohary T (1999). Biovolume calculation for pelagic and benthic microalgae. J. Phycol..

[34] Alverson AJ (2008). Molecular systematics and the diatom species. Protist.

[35] Macgillivary, M. & Kaczmarska, I. *Survey of the Efficacy of a Short Fragment of the rbcL Gene as a Supplemental DNA Barcode for Diatoms*. Vol. 58 (2011). 10.1111/j.1550-7408.2011.00585.x 22092527

[36] Zimmermann J, Jahn R, Gemeinholzer B (2011). Barcoding diatoms: Evaluation of the V4 subregion on the 18S rRNA gene, including new primers and protocols. Org. Divers. Evol..

[37] Kearse M (2012). Geneious basic: An integrated and extendable desktop software platform for the organization and analysis of sequence data. Bioinformatics (Oxford, England).

[38] Katoh K, Standley DM (2013). MAFFT multiple sequence alignment software version 7: Improvements in performance and usability. Mol. Biol. Evol..

[39] Clark K, Karsch-Mizrachi I, Lipman DJ, Ostell J, Sayers EW (2016). GenBank. Nucleic Acids Res..

[40] Guindon S (2010). New algorithms and methods to estimate maximum-likelihood phylogenies: Assessing the performance of PhyML 3.0. Syst. Biol..

[41] Ritz C, Baty F, Streibig JC, Gerhard D (2016). Dose-response analysis using R. PLoS ONE.

[42] Lomas MW, Swain A, Shelton R, Ammerman JW (2004). Taxonomic variability of phosphorus stress in Sargasso Sea phytoplankton. Limnol. Oceanogr..

[43] Yamaguchi H, Yamaguchi M, Adachi M (2006). Specific-detection of alkaline phosphatase activity in individual species of marine phytoplankton. Plankon Benthos Res..

[44] Strickland, J. D. H. & Parsons, T. R. *A Practical Handbook of Seawater Snalysis*. (Fisheries Resrach Board of Canada, 1972).

[45] Bligh EG, Dyer WJ (1959). A rapid method of total lipid extraction and purification. Can. J. Biochem. Phys..

[46] Gašparović B, Kazazić SP, Cvitešić A, Penezić A, Frka S (2015). Improved separation and analysis of glycolipids by Iatroscan thin-layer chromatography–flame ionization detection. J. Chromatogr. A.

[47] Gašparović, B., Kazazić, S. P., Cvitešić, A., Penezić, A. & Frka, S. Corrigendum to “Improved separation and analysis of glycolipids by Iatroscan thin-layer chromatography–flame ionization detection”[J. Chromatogr. A 1409 (2015) 259–267]. (2017). 10.1016/j.chroma.2017.09.038 28951048

[48] Fonda Umani S (2005). Inter-annual variations of planktonic food webs in the northern Adriatic Sea. Sci. Total Environ..

[49] R: A language and environment for statistical computing (R Foundation for Statistical Computing, 2015).

[50] Sprouffske K, Wagner A (2016). Growthcurver: An R package for obtaining interpretable metrics from microbial growth curves. BMC Bioinform..

[51] Schlitzer, R. *Ocean Data View*. http://odv.awi.de (2018).

[52] Smodlaka Tanković M (2018). Experimental evidence for shaping and bloom inducing effects of decapod larvae of *Xantho poressa* (Olivi, 1792) on marine phytoplankton. J. Mar. Biol. Assoc. United Kingdom.

[53] Dyhrman ST (2012). The transcriptome and proteome of the diatom *Thalassiosira pseudonana* reveal a diverse phosphorus stress response. PLoS ONE.

[54] Novak T (2019). Global warming and oligotrophication lead to increased lipid production in marine phytoplankton. Sci Total Environ.

[55] Martin P, Van Mooy BA, Heithoff A, Dyhrman ST (2011). Phosphorus supply drives rapid turnover of membrane phospholipids in the diatom *Thalassiosira pseudonana*. ISME J..

[56] Abida H (2015). Membrane glycerolipid remodeling triggered by nitrogen and phosphorus starvation in *Phaeodactylum tricornutum*. Plant Physiol..

[57] Ivančić I, Degobbis D (1987). Mechanisms of production and fate of organic phosphorus in the northern Adriatic Sea. Mar. Biol..

[58] Hardin G (1960). The competitive exclusion principle. Science.

[59] Hutchinson GE (1961). The paradox of the plankton. Am Nat.

