# Supplementary material for: Morpho-physiological adaptations of Leptocylindrus aporus and L. hargravesii to phosphate limitation in the northern Adriatic
Source: Sci Rep. 2022 Feb 17;12:2687. doi: 10.1038/s41598-022-06062-5 (PMC8854398; doi:10.1038/s41598-022-06062-5)

L. aporus		*L. hargravesii*
P depletion triggers bloom initiation	Lag phase	-
Increased in P depleted conditions	Chain length	Increased in P depleted conditions
High before bloom initiation and during stationary phase → bloom on phosphate pools	Phosphate uptake	High in the early bloom phase and during stationary phase → bloom (initiation) on available dissolved phosphate
High before and during the early bloom phase → organic phosphate important only in bloom initiation and post bloom survival.	Cellular APA	Low before the bloom initiation but increasing during the entire bloom → bloom on organic phosphate resources
Low Km to compete for organic P at low concentrations	AP Km	higher Km to compete for organic P at high concentrations

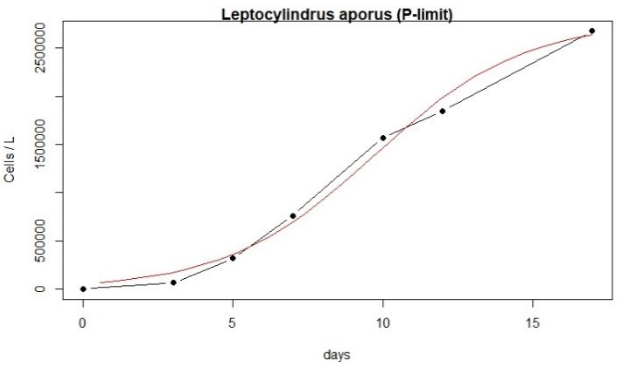

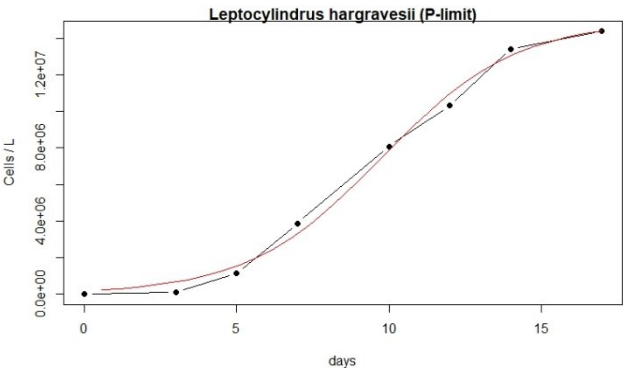

Unbalanced nutrient input results in quick P depletion

Unbalanced nutrient availability results in biogenic P depletion and biogenic release of organic P

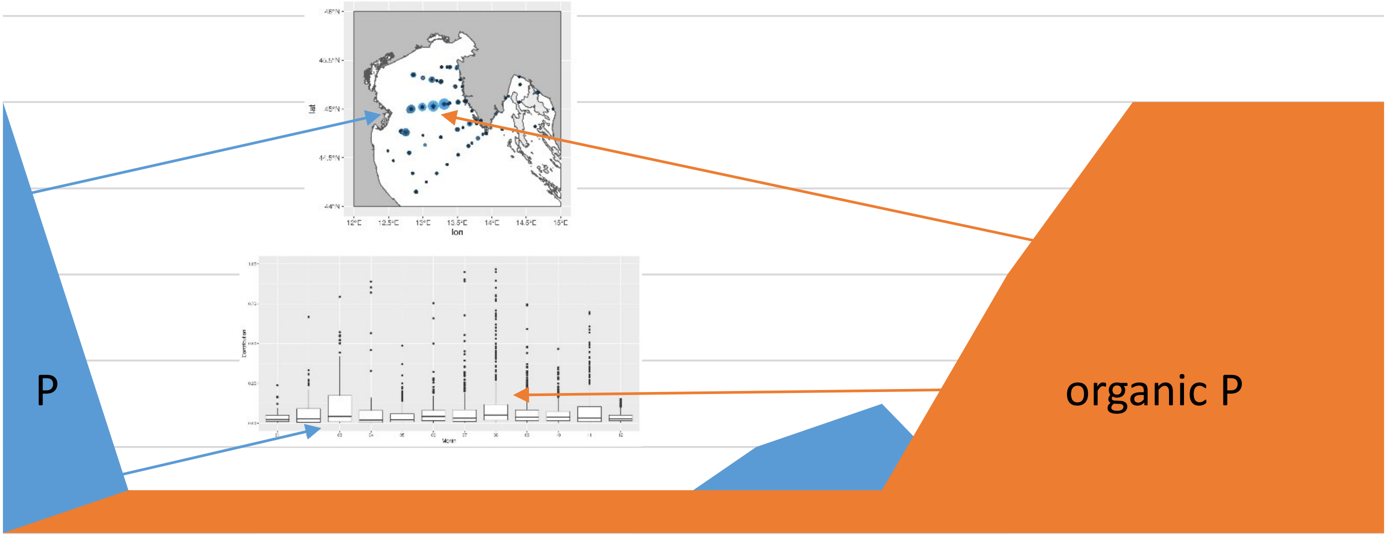

Supplement: Supplementary file 1 — Supplementary Information. [file 41598_2022_6062_MOESM1_ESM.pdf]
